# Establishing a System for Functional Characterization of Full-Length cDNAs of *Camellia sinensis*

**DOI:** 10.3390/ijms20235929

**Published:** 2019-11-25

**Authors:** Lin Lin, Weiwei Cai, Zhenghua Du, Wenjing Zhang, Quanming Xu, Weijiang Sun, Mingjie Chen

**Affiliations:** 1 Fujian Provincial Key Laboratory of Haixia Applied Plant Systems Biology, Horticultural Plant Biology and Metabolomics Center, Haixia Institute of Science and Technology, Fujian Agriculture and Forestry University, Fuzhou 350002, China; michael_lin00@163.com (L.L.); vivivi1025@163.com (W.C.); xiaodu5258@126.com (Z.D.); xquanming@126.com (Q.X.); 2 Anxi College of Tea Science, Fujian Agriculture and Forestry University, Fuzhou 350002, China

**Keywords:** *Camellia sinensis*, full-length cDNA, normalization, rare genes, gene function, binary vector, heterologous expression

## Abstract

Tea (*Camellia sinensis*) is enriched with bioactive secondary metabolites, and is one of the most popular nonalcoholic beverages globally. Two tea reference genomes have been reported; however, the functional analysis of tea genes has lagged, mainly due to tea’s recalcitrance to genetic transformation and the absence of alternative high throughput heterologous expression systems. A full-length cDNA collection with a streamlined cloning system is needed in this economically important woody crop species. RNAs were isolated from nine different vegetative tea tissues, pooled, then used to construct a normalized full-length cDNA library. The titer of unamplified and amplified cDNA library was 6.89 × 10^6^ and 1.8 × 10^10^ cfu/mL, respectively; the library recombinant rate was 87.2%. Preliminary characterization demonstrated that this collection can complement existing tea reference genomes and facilitate rare gene discovery. In addition, to streamline tea cDNA cloning and functional analysis, a binary vector (pBIG2113SF) was reengineered, seven tea cDNAs isolated from this library were successfully cloned into this vector, then transformed into *Arabidopsis*. One FL-cDNA, which encodes a putative P_1B_-type ATPase 5 (*CsHMA5*), was characterized further as a proof of concept. We demonstrated that overexpression of *CsHMA5* in *Arabidopsis* resulted in copper hyposensitivity. Thus, our data demonstrated that this represents an efficient system for rare gene discovery and functional characterization of tea genes. The integration of a tea FL-cDNA collection with efficient cloning and a heterologous expression system would facilitate functional annotation and characterization of tea genes.

## 1. Introduction

Full-length cDNA (FL-cDNA) is the DNA complement to an mRNA sequence that covers the region near the 5′ cap structure to the poly(A) tail [1]. It is essential to identify exon–intron boundaries and gene-coding regions within genomic sequences, and for gene-function analysis at the transcriptional and translational levels [2]. In addition, the FL-cDNA sequences can improve the quality of genome annotation [3], and provide accurate transcription start sites (TSSs), which can increase our understanding of the transcriptional regulation and associated network because transcription factor-binding sites are located around TSSs [4]. Furthermore, FL-cDNA makes it possible to manipulate gene expression in heterologous systems, thus aiding the functional analysis of genes by reverse genetics. Due to these advantages, FL-cDNA technology has been widely applied to the genomic researches of different plant species, including *Arabidopsis* [2], rice [5], soybean [6], corn [7], tomato [8], citrus [3], cotton [9,10], and wheat [11]. However, straight-forward, high-throughput analysis of cDNA libraries is complicated by the differential abundance of various transcripts from any particular cell or tissue; as a result, the identification of rare transcripts from the standard library is often difficult [12]. Thus, a normalized FL-cDNA library is required for rare gene discovery [13]. A normalized cDNA library is that cDNAs derived from abundant mRNAs are greatly reduced, thus originally high- and low-abundance transcripts present in the library at similar frequency.

Tea (*Camellia sinensis*) is rich in bioactive secondary metabolites, and one of the most popular nonalcoholic beverages globally. In 2017, 6 million tons of tea was produced by 20 top production countries, according to the Food and Agriculture Organization of the United Nations (http://faostat.fao.org/). To this day, tea plants have been cultivated in more than fifty countries, and provide massive job opportunities and social wealth [14]. Due to tea’s economic and health importance, many efforts have been made to characterize its agronomic traits. One popular method in tea research is expressed sequence tags (ESTs). Studies of tea transcriptome have provided new insights of the molecular functions of agronomy important genes [15,16,17,18,19]. So far, tea tree genomes from *Camellia sinensis* var. *assamica* (CSA.; Assam type) and *Camellia sinensis* var. *sinensis* (CSS.; Chinese type) have been reported, with 36,951 and 33,932 annotated protein-coding genes, respectively [20,21], and provide valuable resources for the functional characterization of tea genes. Recently, Qiao et al. (2018) applied single molecular real-time (SMRT) sequencing to obtain full-length enriched transcripts for the identification of alternative splicing events [22]. However, a normalized FL-cDNA library in tea tree has not been reported. 

Molecular biology and functional genetic studies have been limited in tea tree, mainly due to two factors: (1) Tea tree is recalcitrant to genetic transformation. The first transgenic tea was reported more than a decade back [23], since then not much progress has been reported even though extensive efforts have been invested. (2) High throughput heterologous expression systems have not been established in tea. To complement and extend previous works, we combined SMART method with duplex-specific nuclease (DSN) treatment, and successfully constructed a normalized tea FL-cDNA library. In order to accelerate the functional analysis of tea genes, a binary vector was reengineered to streamline gene cloning and heterologous expression in *Arabidopsis*. The short generation time and efficient transformation frequency of *Arabidopsis* would accelerate the functional characterization of tea genes. As a proof of the practical application of this FL-cDNA collection, one tea FL-cDNA, which encodes the P_1B_-type ATPase 5 (*CsHMA5*), was cloned and expressed in *Arabidopsis*. The overexpression of *CsHMA5* resulted in copper hyposensitivity, and the phenotypes are consistent with a role of *CsHMA5* as a bona fide tea orthologs of the *Arabidopsis HMA5* gene. The protocols described in this study have thus been proven to be useful for functional characterization of tea genes. 

## 2. Results and Discussion

### 2.1. Construction of a Normalized Full-Length Enriched cDNA Library of Camellia sinensis

In this study, a new normalized EST collection with enrichment of full-length and rare transcripts of tea was successfully generated by applying an integrated method. *Camellia sinensis* cv. ‘*Fuding Dabaicha’* was selected as material for several considerations: (1) it is a national elite germplasm and is widely cultivated in China for the production of green tea, white tea, oolong tea, and black tea; (2) it has been widely used as a maternal line in multiple tea breeding programs in China and produced dozens of new elite germplasms; (3) it is diploid. To maximize the cDNAs included in this library, total RNA was isolated from nine different tissue types, including buds, tender stems, tender leaves, mature leaves, flower buds, flowers, fruit pericarps, roots, and bark. Equal amounts of total RNAs from these tissues were pooled for cDNA library construction. A λ phage (λTriplEx2) was selected as cloning vector since it is not biased toward short insert size. In total, 580 µL of unamplified cDNA library was obtained with a titer of 6.89 × 10^6^ cfu/mL and recombination rate of 87.2%; the titer reached 1.8 × 10^10^ cfu/mL after library amplification. 

**Figure 1:**
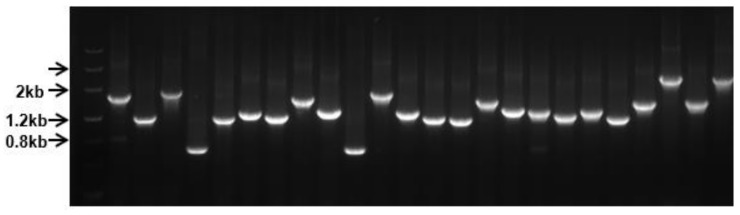
Library insert size evaluation by clonal PCR. Twenty-four white plaques were randomly picked from plates for PCR amplification.

The insert size was evaluated by colony PCR from randomly selected white plaques, which were in the range of 0.8–2.5 kb with medium insertion size of 1.5 kb, as seen in Figure 1. 

### 2.2. Comparison with Tea CSS and CSA Genome

Two tea reference genomes from *Yunkang 10* (*Camellia sinensis* var. *assamica* (CSA.; Assam type)) and *Shuchazao* (*Camellia sinensis* var. *sinensis* (CSS.; Chinese type)) have been published with 36,951 and 33,932 annotated protein-coding genes, respectively [20,21]. The quality of our normalized tea cDNA library was further evaluated by sequencing. Forty-four white plaques were randomly selected and sequenced, after the vector backbone and additional sequences added during cDNA synthesis were trimmed, the obtained sequences of FL-cDNAs were deposited in NCBI database, as seen in Table 1. No redundancy was found, and a total of 44 unigenes were obtained from those 44 clones, including 42 protein-coding RNAs, one long chain noncoding RNA (clone 43, MN027194), and one natural antisense transcript to CSA010175 or TEA005630 (clone 34, MN158199) [24]. Based on gene annotation, these encoded proteins were involved in various cellular processes, including metabolism, transport, protein/RNA modification or transcription factors. Some well-known abundant RNAs, such as chlorophyll a/b binding protein, ribulose-1,5-biphosphate carboxylase, actin, 18S rRNA, alpha-tubulin 10, and ubiquitin [9,25], were not present from this list, suggesting the successful normalization of this cDNA library. 

**Figure 2:**
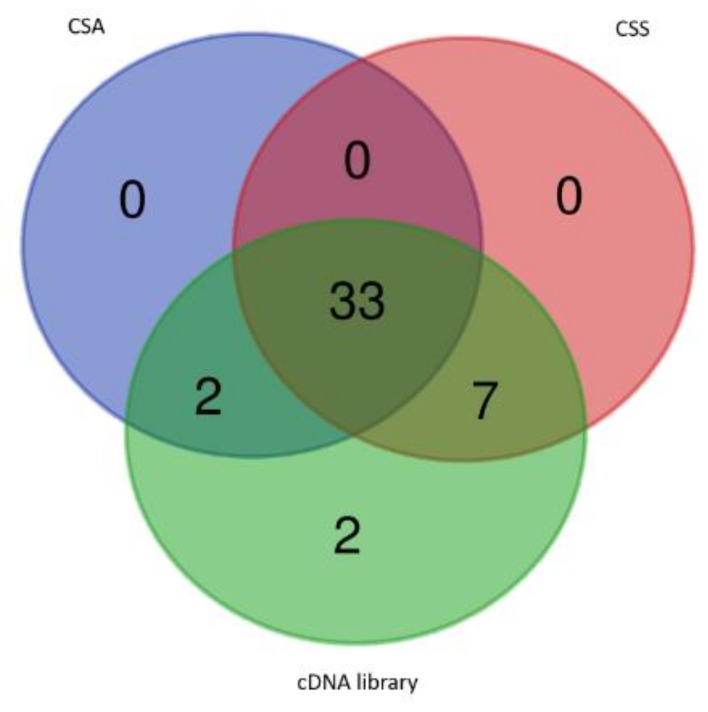
The 44 FL-cDNAs identified in this study showed partial overlap with the *Camellia sinensis* var. *assamica* (CSA.; Assam type) and *Camellia sinensis* var. *sinensis* (CSS.; Chinese type) databases.

These 44 FL-cDNA sequences were used to query the CSS and CSA database, respectively. Thirty-three cDNAs were commonly detected in both databases; seven cDNAs (MK795747, MK795755, MK795757, MK795761, MK795762, MK795764, and MK889354) were found from CSS database but not from CSA database; two cDNAs (MN102719 and MK795766) were found from CSA only. The other two cDNAs, one encoding a natural antisense transcript (clone 34), and the other one encoding a putative long chain noncoding RNA (clone number 43), were not found from CSS and CSA databases, as seen in Figure 2. These observations demonstrated that this normalized tea FL-cDNA library can complement the existing tea genome databases, and identify rare protein coding cDNAs, noncoding RNAs, or natural antisense transcripts. In addition, once this library was extensively sequenced, these sequences’ information can be used to improve the prediction of tea genetic structures.

### 2.3. 5′-UTR and 3′-UTR Analysis

At the whole-genome level, UTRs provide functional specificity to genes in a length-dependent manner [26]. For the calculation of the average length of 5′-UTR and 3′-UTR of tea, three cDNAs were excluded, including clone 29 (incomplete 5′- and 3′-UTR), clone 34 (natural antisense transcript), and clone 43 (noncoding RNA), as seen in Table 1. For the other 41 cDNAs, clone 7 has the shortest (4 bp) 5′-UTR, while clone 40 has the longest (601 bp); clone 42 shows the shortest (39 bp) 3′-UTR, while clone 18 shows and the longest (627 bp). The 5′-UTR lengths for MN027184, MK795746, and MK795755 were only 4, 8, and 11 nt, respectively, as seen in Table 1, raising the question of whether they were truncated during reverse transcription. Thus, they were further verified by RT-PCR. Two flanking forward primers were designed for each cDNA: the first forward primer (F1) ends before the first nucleotide of our identified 5′-UTR sequence, and the second forward primer (F2) starts from the first nucleotide of our identified 5′-UTR sequence; the reverse primers (R) were designed from the respective coding region, as seen in Table S1 A hexamer was used for reverse transcription reaction. If the identified 5′-UTRs were truncated during the library construction, or represent a short version of alternative splicing variant, both F1/R and F2/R primer sets can amplify a predicted fragment; if the identified 5′-UTRs were indeed complete, one would expect that the F2/R primer sets to amplify a predicted fragment, whereas the F1/R primer sets cannot, as seen in Figure S1. 

For MN027184, the annotated CDS from the tea genome database (CSA015215 and TEA028910) has an additional 900 nt before our identified 5′-UTR sequence, as seen in Figure S2A. Both F1/R and F2/R primer sets amplified the predicted size of band, as seen in Figure S2B, suggesting that MN027184 could represent a short isoform of alternative splicing variant. MK795746 showed the same start codon as the annotated CDS from the tea genome database (CSA022245), as seen in Figure S3A, both F1/R and F2/R primer sets amplified a predicted size of band, as seen in Figure S3B, suggesting that 5′-UTR of MK795746 could be truncated. The start codon usage of MK795755 is 13 nt shorter than that of CSS024713, as seen in Figure S4A, both F1/R and F2/R primer sets amplified a predicted size of band, as seen in Figure S4B, suggesting that MK795755 could represent a shorter alternative splicing variant. Global dissection of alternative spicing in tea identified 28,980 AS events [22,26]. 

Based on the validation results above, these three clones were excluded from the calculation of the average length of 5′-UTR, but they are included for the calculation of the average length of 3′-UTR. Our data indicated that the average lengths for the 5′-UTR and 3′-UTR are 128 and 296 bp, respectively. In rice, the average lengths of 5′- and 3′-UTRs are 259 and 469 bp, respectively, but only 155 and 242 bp in *Arabidopsis* [27]. Thus, UTRs in tea tree share higher similarity with *Arabidopsis* than rice. This is unexpected, since the tea genome (3.02 Gb) is 24-fold larger than that of *Arabidopsis* (129 Mb); In addition, as a perennial species tea tree may require more complicated gene regulations compared to annual plant species such as *Arabidopsis* or rice. Thus, it is anticipated that the average length of 5′-UTR and 3′-UTR of tea should be longer than that of *Arabidopsis* or rice. *Arabidopsis* tea tree is a dicot, which may suggest that the 5′-UTR and 3′-UTR from dicot and monocot have been independently evolved after their evolutionary divergence. Since only 41 randomly selected tea FL-cDNAs were used here for UTR length calculation, this tentative estimation requires further validation. The extensive sequencing of this cDNA library in the future would provide more conclusive evidence.

### 2.4. Ectopic Expression of Tea Genes in Arabidopsis

*Arabidopsis* has several advantages for functional analysis of heterologous genes, such as its small size, short generation time, and its high transformation efficiency [28]. Thus, it was used for heterologous expression of tea FL-cDNA. To streamline tea FL-cDNA cloning into the binary vector, the binary vector pBIG2113SF was reengineered to make *Sfi* I cloning sites identical to those of the λ phage vector [29], thus tea FL-cDNAs recovered from this cDNA library can be directly cloned into the pBIG2113SF-M vector with correct orientation for gene expression analysis. 

**Figure 3:**
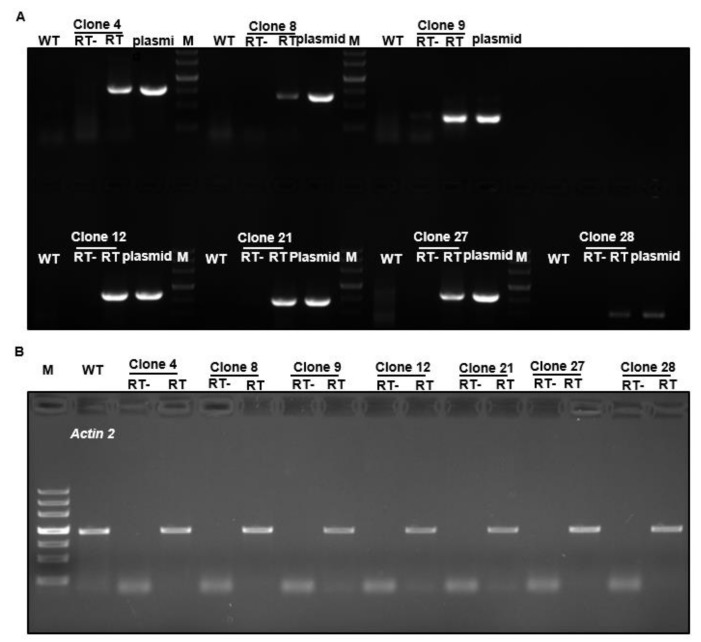
RT-PCR confirmation of transgene expression in Arabidopsis. *Camellia sinensis* FL-cDNAs from clones 4, 8, 9, 12, 21, 27, and 28 were transformed into *Arabidopsis*. (**A**) Total RNA was isolated from respective transgenic plants, tea transgene-specific primer pairs were used for RT-PCR. The respective plasmids were used as positive controls for the PCR. (**B**) *Actin 2* was amplified as the loading control. RT-: no reverse transcriptase added into RT reaction mixture; RT: with reverse transcriptase added into RT reaction mixture.

To validate this cloning and expression system, seven tea FL-cDNAs were selected (clone numbers 4, 8, 9, 12, 21, 27, and 28), as seen in Table 1, and their FL-cDNAs were recovered from their respective plasmids by *Sfi* I digestion, ligated into pBIG2113SF–M vector, and transformed into *Arabidopsis*. RT-PCR analysis of transgenic plants demonstrated that all seven tea genes were successfully transformed and expressed in *Arabidopsis*, as seen in Figure 3. 

### 2.5. Overexpression of a Tea Gene Encoding P_1B_-Type ATPase 5 in Arabidopsis Resulted in Hyposensitivity to Copper

**Figure 4:**
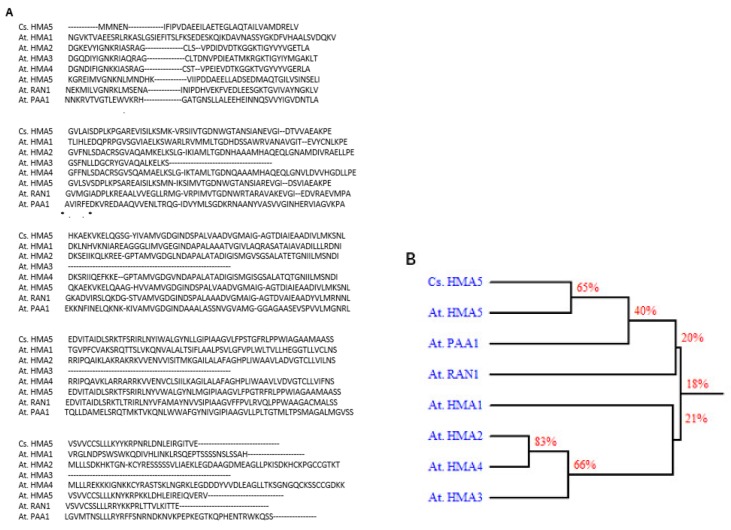
Sequence comparison of *CsHMA5* and *Arabidopsis* orthologs. (**A**) Protein sequence alignment of CsHMA5 with *Arabidopsis* orthologs HMA1 (At4g37270), HMA2 (At4g30110), HMA3 (At4g30120), HMA4 (At2g19110), HMA5 (At1g63440), RAN1 (At5g44790), and PAA1 (At4g33520). (**B**) Phylogenetic tree of the amino acid sequence of CsHMA5 and the seven *Arabidopsis* HMA protein sequences.

**Figure 5:**
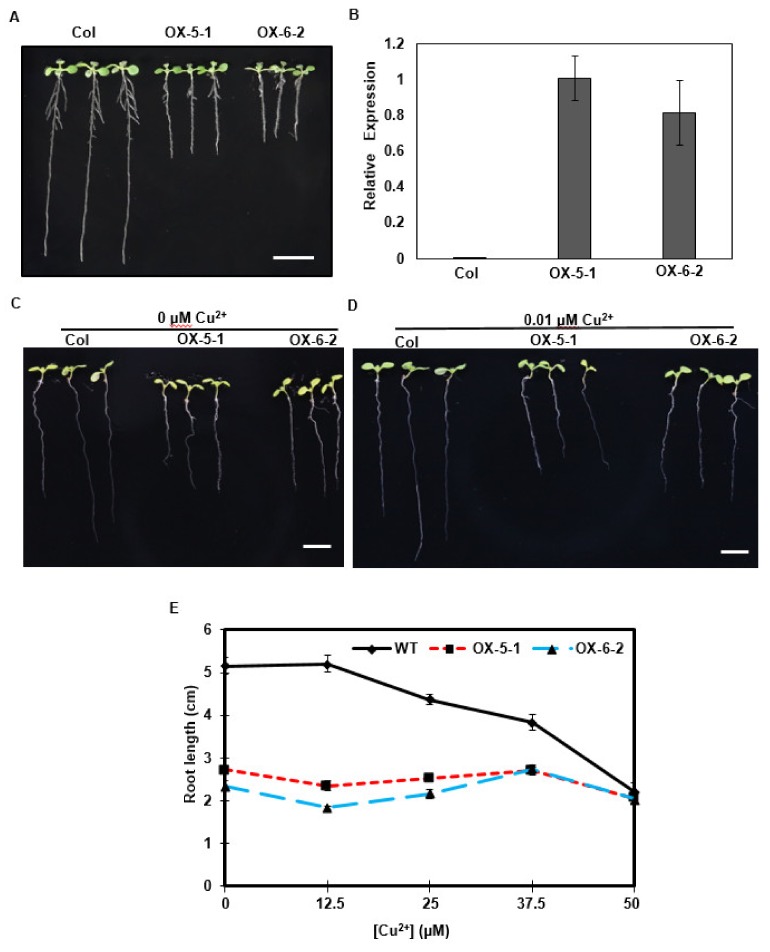
Overexpression of *CsHMA5* in *Arabidopsis* reduced copper sensitivity. (**A**) *CsHMA5* overexpressors showed short roots on standard 0.5× MS medium. (**B**) RT-qPCR analysis of the expression levels of control and two *CsHMA5* overexpressors. (**C**,**D**) *Col* and two *CsHMA5* overexpressors (OX-5-1 and OX-6-2) were germinated on synthetic 1× MS medium without copper or low copper concentration (0.01 µM). (**E**) *Col* and two *CsHMA5* overexpressors (OX-5-1 and OX-6-2) were germinated on standard 0.5× MS medium supplemented with 12.5–50 µM copper. The seeds were germinated vertically under continuous light, the root lengths were measured from 10-day-old seedlings. Data are expressed as mean ± SE (*n* = 28). Scale Bar = 1 cm.

Clone 8 corresponds to a 1183 nt transcript with a 209-nt 5′-UTR and 248-nt 3′-UTR, and encodes a deduced 25.6 kDa protein of 241 amino acids. Its amino acid sequence showed high similarity to *Arabidopsis* HMA5 protein, as seen in Figure 4, indicating that clone 8 encodes a tea ortholog of *AtHMA5*. Thus, we renamed the clone 8 cDNA as *CsHMA5*. *CsHMA5* was transformed into *Arabidopsis*. Several hygromycin-resistant homozygous lines were identified, and two overexpression lines (OX-5-1 and OX-6-2) were characterized further. When germinated on standard 0.5× MS solid medium, transgenic plants showed shorter primary and lateral roots than that of WT control, as seen in Figure 5A. RT-qPCR results demonstrated that *CsHMA5* was highly and similarly expressed from both overexpressors, but was undetected in the WT control, as seen in Figure 5B. HMA5 mainly catalyzes copper export from the root cells in *Arabidopsis*. The standard 1× MS medium contains 0.1 µM copper. To test if the short root phenotype of the overexpressors was a result of their copper hypersensitivity, WT and two overexpressors were germinated on synthetic 1× MS medium without copper or low copper (0.01 µM). Under both growth conditions, the root lengths of OX-5-1 and OX-6-2 were still shorter than that of control, as seen in Figure 5C,D. These data suggested that overexpression of *CsHMA5* in *Arabidopsis* did not result in copper hypersensitivity. To test if CsHMA5 overexpressors could show altered response to higher concentrations of copper, seeds of WT and two *CsHMA5* overexpressors were germinated on standard 0.5× MS medium supplemented with 12.5–50 µM copper. The exogenously supplementation of copper into standard MS medium would increase its free copper concentration accordingly. After plant growth for 10 days, WT root length from control plate was almost two-fold longer as that of *CsHMA5* overexpressors. With the increasing concentration of copper, WT root length became gradually shorter, and this growth inhibition became more evident when exogenously supplied copper reached above 12.5 µM.; in contrast, the root growth from *CsHMA5* overexpressors was not affected by as high as 37.5 µM copper, as seen in Figure 5D. These observations demonstrated that overexpression of *CsHMA5* in *Arabidopsis* led to copper hyposensitivity.

In *Arabidopsis*, *HMA5* is primarily expressed in roots, and strongly and specifically induced by copper in whole plants [30]. To further investigate the mechanisms of how *CsHMA5* overexpression rendered copper sensitivity of transgenic plants, 16 genes from copper signaling pathways were selected, and their expression levels in the root tissues were compared between WT and *CsHMA5* overexpressors. We found that the expression levels of the endogenous *AtHMA5* from *CsHMA5* overexpressors were not affected, as seen in Figure S5A–F. However, the expression levels of *COPT3* and *COX17-1* in transgenic lines were significantly higher than that of WT, while *COPT5*, *RAN1*, and *PAA2* showed significantly lower expression levels than that of WT, as seen in Figure S5B. The expression levels for the rest 10 genes, including *HMA1*, *PPA1*, *CPOT1*, *CPOT2*, *CPOT4*, *COX17-2*, *ZIP2*, *ZIP4*, *ATX1*, and *CCS1*, were not significantly affected, as seen in Figure S5G–P. *COX17-1* is a soluble protein located in the mitochondrial intermembrane space and participates in the transfer of copper for cytochrome oxidase assembly [31], the expression of which is induced by high copper concentrations [32]. *COPT5* is a tonoplast copper exporter responsible for the transport of copper ions from root to reproductive organs [33]. *RAN1* plays an essential role in the biogenesis of the ethylene receptors and copper homeostasis [34]. *PAA2* is a chloroplast copper transporter of the thylakoid membrane [35]. These observations suggested that these changes in gene expression might affect copper transport and interorgan copper reallocation. Genetic analysis demonstrated that knockout *HMA5* in *Arabidopsis* results in hypersensitivity to copper. In contrast, expression of *SvHMA5II* in *Arabidopsis* increases copper tolerance [36]. In this study, we demonstrated that overexpression of *CsHMA5* in *Arabidopsis* resulted in hyposensitivity to copper, as seen in Figure 5. These data demonstrated that *Arabidopsis* can be used as a heterologous host to study the functions of tea genes.

## 3. Materials and Methods

### 3.1. Plant Materials

Tea plants (*Camellia sinensis* (L.) O. Kuntze cv. ‘*Fuding Dabaicha*’) were grown in the tea garden of Fujian Agriculture and Forestry University. Nine different tissues, including roots, tender stems, tender leaves, mature leaves, flower buds, flowers, fruit pericarps, and bark, were collected on September 2015.

### 3.2. Total RNA Extraction

Total RNA was isolated by using modified CTAB method [37]. A total of 1.5 g tissue was ground into fine powder by mortar and pestle in the presence of liquid nitrogen and a small amount of polyvinyl pyrrolidone (PVP, A610436, sangon, shanghai, China); the powder was transferred into 50 mL tube containing 9 mL of CTAB solution and 450 µL of β-mercaptoethanol(80076928, Sinopharm chemical reagent, shanghai, China), heated at 65 °C water bath for 20 min, then centrifuged at 12,000× *g* for 10 min at 4 °C. The supernatant was transferred into a fresh tube containing equal volume of prechilled phenol:chloroform:isopropanol (25:24:1, *v*/*v*/*v*) ( P1012, Solabio, Beijing, China), mixed well, incubated on ice water bath for 10 min, then centrifuged at 12,000× *g* for 10 min at 4 °C. The above extraction steps were repeated, and the supernatant was then transferred into fresh tube containing equal volume of prechilled chloroform:isopropanol (24:1, *v*/*v*) ( P1014, Solabio, Beijing, China), mixed well, and centrifuged at 12,000× *g* for 15 min at 4 °C. The supernatant was once again transferred into a fresh tube containing half volume of prechilled 8 M LiCl (A100416, sangon, Shanghai, China) and 1% β-mercaptoethanol, and then stored at −20 °C overnight. The next day, the mixture was spun at 12,000× *g* for 30 min at 4 °C, the supernatant was discarded, and the pellet was rinsed with prechilled 75% ethanol, air dried for 10 min, and dissolved in 200–500 µL DEPC-treated water, as seen in Figure S6.

### 3.3. mRNA Isolation

A 500 µg RNA aliquot from each tissue type was pooled together, then used to isolate mRNA by applying PolyATract mRNA Isolation System (Promega, Madison, WI, USA). The isolated mRNA was eluted into 1 mL of RNase-free water, as seen in Figure S7.

### 3.4. cDNA Synthesis

The first cDNA strand was synthesized from 1 µg of purified mRNA by using SMART cDNA library construction kit (Clontech, Mountain view, CA, USA), SMART IV Oligonucleotide and CDS-4M adapter were used for amplification, as seen in Figure S8. dsDNA was synthesized by using Advantage 2 PCR kit (Clontech, Mountain view, CA, USA), PCR Primer M1 and CDS-4M adapter were used as primers, as seen in Figure S9. The amplified ds cDNA was purified by QIAquick PCR Purification Kit (Qiagen, Hilden, Germany). 

### 3.5. cDNA Denaturation, Hybridization, and DSN Treatment for Normalization

DSN (Evrogen, Moscow, Russia) isolated from Kamchatka crab was used for normalization. A total of 0.6–1.2 µg purified ds cDNA, 4 µL of 4× hybridization buffer, and an appropriate volume of sterile RNase-free water was combined to make total volume of 16 µL, mixed well, and spun briefly. A 4 µL aliquot of the reaction mixture was taken into each of the four appropriately labeled sterile PCR tubes, the reaction mixture was overlaid with a drop of mineral oil, and the tubes were centrifuged for 2 min at maximum speed in a microcentrifuge. The tubes were incubated in a thermal cycler at 98 °C for 2 min, followed by incubation at 68 °C for 5 h. Shortly before the end of the hybridization procedure, two DSN dilutions were prepared in two sterile tubes to final DSN concentrations of 0.5 and 0.25 U/µL, then placed on ice. The DSN master buffer was preheated at 68 °C for 3–5 min, 5 µL of the hot DSN master buffer was added to each tube containing hybridized cDNA, and the tube was briefly spun in a microcentrifuge and returned quickly to the thermal cycler, and incubated at 68 °C for 10 min. Then 0, 0.25, 0.5, and 1 U DSN were added into each tube, then immediately returned to the thermal cycler, incubated at 68 °C for 25 min. A total of 5 µL of DSN stop solution was mixed in the tubes and spun briefly, incubated in the thermal cycler at 68 °C for 5 min, and then the tubes were placed on ice. A total of 25 µL of sterile RNase-free water was mixed in each tube and spun briefly, then placed on ice, as seen in Figure S10. 

### 3.6. Amplification of Normalized cDNA 

Well-normalized cDNA was amplified by Advantage 2 PCR Kit (Clontech, CA, USA). PCR Primer M1 was used for amplification, as seen in Figure S11. The amplified ds cDNA was purified by using QIAquick PCR Purification Kit (Qiagen, Hilden, Germany), and eluted with 79 µL of sterile RNase-free water.

### 3.7. Sfi I Digestion and cDNA Size Fractionation

Purified cDNA was digested by *Sfi* I (R0123V, NEB, Ipswich, MA, USA) at 50 °C for 2 h, then size fractioned by using CHROMA SPIN TM-400 column. The first four fractions (lanes 5–8) were collected and pooled into a clean tube, as seen in Figure S12, precipitated with 95% ethanol at −20 °C overnight, air dried, and the pellet was resuspended in 7 μL of deionized H_2_O. The *Sfi* I-digested cDNA is then ready to be ligated into the *Sfi* I-digested, dephosphorylated λTriplEx2 vector (Clontech, CA, USA).

### 3.8. cDNA Ligation into λTriplEx2 Vector

To ligate *Sfi* I digested cDNA to λTriplEx2 vector (500 ng), three different ligations with varying insert to vector ratio were performed, as seen in Figure S13. The ligation was conducted at 16 °C overnight, then heated in a 65 °C water bath for 5 min to deactivate the T4 DNA ligase. 

### 3.9. Package Ligation Mixture

To package into the λTriplEx2 vector, 25 µL of MaxPlax Lambda Packaging Extracts (Epicentre, Madison, WI, USA) was added into above ligation mixture, the detailed packaging procedure followed manufacturer’s instructions. 

### 3.10. Titering the Unamplified Library and Determination of the Rate of Recombination

To titer the unamplified library, the library was diluted to 10^−2^–10^−3^ with phage dilution buffer, then a 100 µL aliquot was mixed with 100 µL *E. coli* XL1-blue cell, incubated at 37 °C for 15 min, and then plated onto LB/MgSO_4_ medium. To determine the recombinant rate, before plating onto LB/MgSO_4_ medium, 3.0 mL of melted LB/MgSO_4_ top agar (45 °C) containing 75 µL IPTG Fand 75 µL X-gal was added and mixed well. The plates were incubated at 37 °C for 16–18 h, then plaque numbers were counted. The recombinant rate was expressed as the ratio of white (recombinant) to blue (nonrecombinant) plaques.

### 3.11. Library Amplification and Titering 

The library was amplified and titered according to product manual. 

### 3.12. Converting λTriplEx2 to pTriplEx2 Plasmid

A well-isolated positive plaque from secondary or tertiary screening plate was picked, coincubated with 200 µL of *E. coli* BM25.8 at 31 °C for 30 min without shaking, and 1–10 µL of infected cell suspension was spread onto LB/carbenicillin plate to obtain isolated colonies. 

### 3.13. cDNA Sequencing

Plasmid DNAs were prepared from randomly selected 44 white clones and sequenced by using standard ds-DNA sequencing protocols. The forward and reverse sequencing primers were: 5′-TAATACGACTCACTATAGGGC-3′; and 5′-CTCGGGAAGCGCGCCATTGTG-3′, respectively. The 5′-UTR and 3′-tail sequences were determined from individual cDNA. The CDS sequence was used to query the CSA and CSS genomes using BLAST [38].

### 3.14. Binary Vector pBIG2113SF-M Construction

Binary vector pBIG2113SF was selected to express tea FL-cDNAs in planta [29]. The *Sfi* IA and *Sfi* IB cutting sites of pBIG2113SF vector are different from λTriplEx2 vector and were re-engineered to make them same as λTriplEx2 vector. As a first step, the *Sfi* IA and *Sfi* IB cutting sites from vector U12445 were first modified by using Fast Site-Directed Mutagenesis Kit (TIANGEN, Beijing, China). The primer sequences for *Sfi* IA mutagenesis were: 5′-ATCTGGAATTCGGCCATTACGGCCAGAAGGAGATA-3′; and 5′-TATCTCCTTCTGGCCGTAATGGCCGAATTCCAGAT-3′. The primer sequences for *Sfi* IB mutagenesis were: 5′-AAAGTGCCTAAGGCCGCCTCGGCCGTCGACTAGAA-3′; and 5′-TTCTAGTCGACGGCCCGGAGGGCCTTAGGCACTTT-3′. The new U12445 vector with altered *Sfi* IA and *Sfi* IB recognition sequence was renamed as U12445M. 

The DNA fragment spanning *Sfi* IA and *Sfi* IB from U12445M vector was amplified by using following pair of primers: 5′-TACAACTACATCTAGAGTTATCTGGAATTCGGCCATTA-3′; and 5′-CCGGGGATCCTCTAGAAATTCTAGTCGACGGCCGAG-3′. The PCR products were separated by agarose gel electrophoresis and purified by using E.Z.N.A.^®^ Gel Extraction Kit (Omega Bio-tek, Norcross, GA, USA), then cloned into pBIG2113SF vector by gene fusion method. The engineered pBIG2113SF vector with new *Sfi* IA and *Sfi* IB sites was renamed as pBIG2113SF-M. 

### 3.15. Cloning Tea FL-cDNAs into pBIG2113SF-M Vector

Individual clones from the tea cDNA library were converted into pTriplEx2 plasmid, the tea FL-cDNA insert was recovered from the plasmid by *Sfi* I digestion at 50 °C for 3 h, purified by gel extraction kit (Omega Bio-tek, Norcross, GA, USA), and then ligated into *Sfi* I sites of pBIG2113SF-M vector by T4 DNA ligase (New England Bio Labs, Beverly, MA, USA) at 16 °C overnight. In this report, seven tea FL-cDNAs were cloned into the pBIG2113SF-M vector. 

### 3.16. Transforming Tea FL-cDNA into Arabidopsis

Individual recombinant pBIG2113SF-M vectors containing tea FL-cDNA insert were introduced into *Agrobacterium tumefaciens* GV3101 and transformed into *Arabidopsis thaliana Columbia* by the floral dip method. The T1 seeds were harvested and screened in 0.5× MS medium containing 50 μg mL^−1^ hygromycin. Resistant seedlings were transferred into soil pots, and T2 seeds were harvested from individual plants. T2 seeds were screened in 0.5× MS medium containing 50 μg mL^−1^ hygromycin. Six resistant seedlings per line were transferred into soil pot, and T3 seeds were harvested individually. Homozygote transgenic T3 seeds were identified and used for further characterization. Tea gene-specific primers were used to confirm transgene expression by RT-PCR, as seen in Table S1. 

### 3.17. Root Length Measurement and Gene Expression Analysis

The standard 0.5× MS medium was prepared from Murashige & Skoog basal medium vitamins (Phyto Technology Laboratories, Shawnee Mission, KS, USA). A total of 2.2 g of MS basal medium and 0.5 g of MES were dissolved in 1 L of ddH_2_O, the pH adjusted to 5.7, and 8 g of agar powder (Solarbio, Beijing, China) was added, then copper sulfate was supplemented to specified concentrations. The composition of the synthetic copper deficiency 1× MS medium was (per liter of medium): 1650 mg NH_4_NO_3_, 1900 mg KNO_3_, 440 mg CaCl_2_.2H_2_O, 370 mg MgSO_4_.7H_2_O, 170 mg KH_2_PO_4_, 6.2 mg H_3_BO_3_, 16.9 mg MnSO_4_.H_2_O, 8.6 mg ZnSO_4_.H_2_O, 0.83 mg KI, 0.25 mg NaMoO_4_.2H_2_O, 0.025 mg CoCl_2_.6H_2_O, 27.8 mg FeSO_4_.7H_2_O, 37.3 mg Na_2_EDTA.2H_2_O, 103 mg B5 vitamin mix, 15,000 mg sucrose, 8000 mg agar. All the macrosalts and sucrose used in this study were analytical grade. Homozygote transgenic T3 seeds were germinated vertically on growth medium under continuous light (100 µmol photon m^−2^ s^−1^). After plant growth for 10 days, root lengths were measured by using Image J software (version 1.48, NIH, Washington, DC, USA). Data were expressed as mean ± SE (*n* = 28). After root length measurement, root tissues were harvested to isolate total RNA. One microgram of total RNA was reverse transcribed using PrimeScript™ RT Reagent Kit (Perfect Real Time) (TaKaRa, Japan) and a mixture of oligo (dT)_12–18_ primer and random hexamers were added. The RT products were diluted three-fold before use as qPCR templates. The primer pairs for copper signaling genes are listed in Table S2. qPCR was performed using a SYBR Premix Ex Taq™ II (TliRNase H Plus) kit (TaKaRa, Dalian, China) and CFX Connect™ Optics Module (Bio-Rad, Pleasanton, CA, USA) according to the manufactures’ instructions. *Arabidopsis ADAPTOR PROTEIN-2 MU-ADAPTIN* (*AP2M*, At5g46630) was used as loading control. Each reaction was performed in triplicate along with an internal control reaction. Relative gene expression levels were calculated according to the 2^−ΔΔ*C*t^ comparative CT method [39].

### 3.18. Bioinformatic Analysis

Protein sequence comparison and the phylogenetic tree construction of *Arobidopsis* P_1B_-type ATPases and *CsHMA5* were done by using DNAMAN software (version: 7.0.2.176, Lynnon Corporation, San Ramon, CA, USA).

## 4. Conclusions

Tea is an obligate out-breeding plant species, which makes its genome highly heterozygous. Its seeds contain genome from two different tea germplasms. To avoid RNA contamination from other tea germplasms, in this study, only vegetative tissues from a single cultivar ‘*Fuding Dabaicha*’ were used to make the RNA pool. As a result, our cDNA library also excludes those genes that are specifically expressed in tea seeds.

There are several procedures to make a FL-cDNA library, mostly based on the mRNA cap structure [40,41]. These methods require high quantities of starting material and complicated multistep manipulations of the cap structure of mRNA and cDNA intermediates, which often cause mRNA degradation [42]. In this study, we applied the SMART™ method, which has been demonstrated to yield longer average ORF length [42,43]. To decrease the prevalence of clones from abundant transcripts, DSN from the Kamchatka crab was applied to specifically cleave ds-DNA in both DNA-DNA and DNA-RNA duplexes, which allowed us to separate and then PCR amplified the normalized ss- fraction. We also used λ phage (λTriplEx2) as vector for this library construction. Compared with plasmid vectors, the λ phage can accommodate cDNAs in broad ranges of sizes and shows high efficiency for long cDNA fragments. In contrast, plasmid vectors are often biased toward short cDNA fragments [44]. Furthermore, λ phage can be easily converted into plasmid for downstream applications.

Here we performed a small-scale trial and demonstrated that this FL-cDNA library is a valuable resource for new tea gene discovery and functional characterization. A more depth sequencing of this library would be simply a matter of cost. The establishment of easy tea gene cloning in combination with highly efficient *Arabidopsis* transformation will obviate the technical hurdle of direct tea transformation, thus facilitating functional analysis of tea genes. This system has the potential to be scaled up by using pooled inserts for cloning and transformation, and a large transgenic population can be easily generated for phenotypic observation. Once phenotypes were scored and corresponding tea transgenes identified, the functions of corresponding tea genes can be elucidated. Such strategies have been applied to study gene functions of model plant species, such as *Arabidopsis* and rice [45,46], and should be more applicable to nonmodel plant species such as tea tree.

## Figures and Tables

**Table 1:** Forty-four unigenes identified from the tea cDNA library.

Clone Number	Accession Number	CSA Number	CSS Number	5′UTR (bp)	ORF (bp)	3′UTR (bp)	Gene Annotation
**1**	MN027182	CSA023243	TEA018739	22	654	335	polyadenylate-binding protein 2 isoform X1
**2**	MK795745	CSA033654	TEA031121	33	912	505	α-1,3/1,6-mannosyltransferase ALG2-like
**3**	MK795746	CSA022245	TEA026028	8	828	308	protein COFACTOR ASSEMBLY OF COMPLEX C SUBUNIT B CCB2, isoform X7
**4**	MN027183	CSA027624	TEA005502	73	1353	309	vacuolar protein sorting-associated protein 9A-like
**5**	MK795747	nd	TEA018613	194	1140	254	F-box protein At3g07870-like
**6**	MK795748	CSA033750	TEA021240	81	459	459	signal peptidase complex catalytic subunit SEC11A-like
**7**	MN027184	CSA015215	TEA028910	4	1158	213	pentatricopeptide repeat-containing protein At5g50390, chloroplastic
**8**	MK795749	CSA019466	TEA000616	209	726	248	Probable copper-transporting ATPase HMA5
**9**	MN027185	CSA017490	TEA009722	93	315	280	uncharacterized LOC114266360 (LOC114266360)
**10**	MK889351	CSA017486	TEA002539	102	768	276	thioredoxin-like 2, chloroplastic
**11**	MK795750	CSA028127	TEA008577	157	579	258	protein RER1B-like
**12**	MK795751	CSA028739	TEA024852	32	963	305	probable serine/threonine-protein kinase At1g54610
**13**	MN125540	CSA004353	TEA017437	45	1800	210	LOW quality protein: BTB/POZ domain-containing protein At3g08570-like
**14**	MN102719	CSA012903	nd	237	660	432	LOW QUALITY PROTEIN: DExH-box ATP-dependent RNA helicase DExH9-like
**15**	MK795752	CSA004217	TEA003309	66	555	272	protein EI24 homolog
**16**	MK889352	CSA008914	TEA006217	310	477	260	LOW QUALITY PROTEIN: histone-lysine N-methyltransferase CLF
**17**	MN027187	CSA015641	TEA002353	61	555	181	acid phosphatase 1
**18**	MK795753	CSA030107	TEA029671	23	483	627	ROOT primordium defective 1
**19**	MK795754	CSA023247	TEA002601	206	1149	252	E3 ubiquitin-protein ligase SIS3-like
**20**	MK795755	nd	TEA024713	11	1059	194	uncharacterized protein LOC114322803 isoform X2
**21**	MK795756	CSA012514	TEA005305	19	954	200	2-oxoglutarate-dependent dioxygenase AOP3-like
**22**	MK795757	nd	TEA001038	31	1374	351	protein farnesyltransferase subunit beta isoform X2
**23**	MK795758	CSA018660	TEA017439	28	1014	433	protein XAP5 CIRCADIAN TIMEKEEPER
**24**	MK889353	CSA006900	TEA028663	383	591	259	cinnamoyl-CoA reductase 1-like isoform X1
**25**	MN027188	CSA015703	TEA028160	147	648	403	uncharacterized protein LOC114291801
**26**	MK795759	CSA028933	TEA016139	60	483	220	40S ribosomal protein S11
**27**	MK795760	CSA021019	TEA026343	25	1077	142	methylmalonate-semialdehyde dehydrogenase (acylating), mitochondrial-like isoform X3
**28**	MK795761	nd	TEA030658	88	516	332	Universal stress protein A-like protein isoform X1
**29**	MN027193	CSA035528	TEA027668		1013		cylicin-1-like isoform X2
**30**	MN027189	CSA008021	TEA020012	37	876	224	B3 domain-containing transcription factor VRN1-like
**31**	MK795762	nd	TEA005586	81	828	213	uncharacterized LOC114272112, transcript variant X2,
**32**	MK795763	CSA019133	TEA015571	143	618	278	uncharacterized protein LOC114308887
**33**	MK795764	nd	TEA019546	20	918	177	uncharacterized LOC114312832
**34**	MN158199	nd	nd				Natural antisense RNA to CSA010175 or TEA005630
**35**	MK795765	CSA023599	TEA024700	338	807	354	uncharacterized LOC114281519, transcript variant X2
**36**	MK795766	CSA001233	nd	43	480	302	Uncharacterized protein LOC104594327
**37**	MK795767	CSA031667	TEA000191	363	666	262	uncharacterized protein LOC114256570
**38**	MN027190	CSA000092	TEA023002	11	645	341	uncharacterized protein At4g15545-like
**39**	MN027191	CSA017175	TEA009315	152	879	540	uncharacterized protein LOC114261191 isoform X2
**40**	MN027192	CSA029843	TEA002496	601	750	305	phospholipase A1-IIgamma-like (LOC114274378)
**41**	MK795768	CSA009902	TEA027481	66	651	256	uncharacterized LOC114314960
**42**	MK795769	CSA026559	TEA003389	243	768	39	Uncharacterized protein
**43**	MN027194	nd	nd				Noncoding RNA
**44**	MK889354	nd	TEA023793	39	654	317	RNA-binding protein 48-like isoform X2

nd: not detected.

## Supplementary Materials

The following are available online at https://www.mdpi.com/1422-0067/20/23/5929/s1.

## References

[1] Suzuki Y., Yoshitomo-Nakagawa K., Maruyama K., Suyama A., Sugano S. (1997). Construction and characterization of a full length-enriched and a 5′-end-enriched cDNA library. Gene.

[2] Seki M., Narusaka M., Kamiya A., Ishida J., Satou M., Sakurai T., Nakajima M., Enju A., Akiyama K., Oono Y. (2002). Functional annotation of a full-length Arabidopsis cDNA collection. Science.

[3] Marques M.C., Alonso-Cantabrana H., Forment J., Arribas R., Alamar S., Conejero V., Perez-Amador M.A. (2009). A new set of ESTs and cDNA clones from full-length and normalized libraries for gene discovery and functional characterization in citrus. BMC Genom..

[4] Makita Y., Shimada S., Kawashima M., Kondou-Kuriyama T., Toyoda T., Matsui M. (2015). MOROKOSHI: Transcriptome database in sorghum bicolor. Plant Cell Physiol..

[5] Kikuchi S., Satoh K., Nagata T., Kawagashira N., Doi K., Kishimoto N., Yazaki J., Ishikawa M., Yamada H., Ooka H. (2003). Collection, mapping, and annotation of over 28,000 cDNA clones from japonica rice. Science.

[6] Umezawa T., Sakurai T., Totoki Y., Toyoda A., Seki M., Ishiwata A., Akiyama K., Kurotani A., Yoshida T., Mochida K. (2008). Sequencing and analysis of approximately 40,000 soybean cDNA clones from a full length-enriched cDNA library. DNA Res..

[7] Soderlund C., Descour A., Kudrna D., Bomhoff M., Boyd L., Currie J., Angelova A., Collura K., Wissotski M., Ashley E. (2009). Sequencing, mapping, and analysis of 27,455 Maize full-length cDNAs. PLoS Genet..

[8] Aoki K., Yano K., Suzuki A., Kawamura S., Sakurai N., Suda K., Kurabayashi A., Suzuki T., Tsugane T., Watanabe M. (2010). Large-scale analysis of full-length cDNAs from the tomato (Solanum lycopersicum) cultivar Micro-Tom, a reference system for the Solanaceae genomics. BMC Genom..

[9] Lin M., Lai D., Pang C., Fan S., Song M., Yu S. (2013). Generation and analysis of a large-scale expressed sequence tag database from a full-length enriched cDNA library of developing leaves of *Gossypium hirsutum* L.. PLoS ONE.

[10] Zhang W., Zhang H., Qi F., Jian G. (2016). Generation of transcriptome profiling and gene functional analysis in *Gossypium hirsutum* upon *Verticillium dahliae* infection. Biochem. Biophys. Res. Commun..

[11] Ogihara Y., Mochida K., Kawaura K., Murai K., Seki M., Kamiya A., Shinozaki K., Carninci P., Hayashizaki H., Shin-I T. (2004). Construction of a full-length cDNA library from young spikelets of hexaploid wheat and its characterization by large-scale sequencing of expressed sequence tags. Genes Genet. Syst..

[12] Carninci P., Shibata Y., Hayatsu N., Sugahara Y., Shibata K., Itoh M., Konno H., Okazaki Y., Muramatsu M., Hayashizaki Y. (2000). Normalization and subtraction of CAP-trapper-selected cDNAs to prepare full-length cDNA libraries for rapid discovery of new genes. Genome Res..

[13] Zhulidov P.A., Bogdanova E.A., Shcheglov A.S., Shagina I.A., Vagner L.L., Khazpekov G.L., Kozhemiako V.V., Luk’ianov S.A., Shagin D.A. (2005). A method for the preparation of normalized cDNA libraries enriched with full-length sequences. Russ. J. Bioorg. Chem..

[14] Chen L., Apoaroliswa Z., Chen Z.M. (2012). Global Tea Breeding: Achievement, Challenges and Perspectives.

[15] Di T., Zhao L., Chen H., Qian W., Wang P., Zhang X., Xia T. (2019). Transcriptomic and metabolic insights into the distinctive effects of exogenous melatonin and gibberellin on terpenoid synthesis and plant hormone signal transduction pathway in *Camellia sinensis*. J. Agric. Food Chem..

[16] Guo Y., Zhu C., Zhao S., Zhang S., Wang W., Fu H., Li X., Zhou C., Chen L., Lin Y. (2019). *De novo* transcriptome and phytochemical analyses reveal differentially expressed genes and characteristic secondary metabolites in the original oolong tea (*Camellia sinensis*) cultivar ‘Tieguanyin’ compared with cultivar ‘Benshan’. BMC Genom..

[17] Gao Y., Zhao M., Wu X., Li D., Borthakur D., Ye J., Zheng X., Lu J. (2019). Analysis of differentially expressed genes in tissues of *Camellia sinensis* during dedifferentiation and root redifferentiation. Sci. Rep..

[18] Xin Z., Ge L., Chen S., Sun X. (2019). Enhanced transcriptome responses in herbivore-infested tea plants by the green leaf volatile (Z)-3-hexenol. J. Plant Res..

[19] Wu L., Fang Z., Lin J., Sun Y., Du Z., Guo Y., Liu J., Liang Y., Ye J. (2019). Complementary iTRAQ proteomic and transcriptomic analyses of leaves in tea plant (*Camellia sinensis* L.) with different maturity and regulatory network of flavonoid biosynthesis. J. Proteome Res..

[20] Xia E., Zhang H., Sheng J., Li K., Zhang Q., Kim C., Zhang Y., Liu Y., Zhu T., Li W. (2017). The tea tree genome provides insights into tea flavor and independent evolution of caffeine biosynthesis. Mol. Plant.

[21] Wei C., Yang H., Wang S., Zhao J., Liu C., Gao L., Xia E., Lu Y., Tai Y., She G. (2018). Draft genome sequence of *Camellia sinensis* var. sinensis provides insights into the evolution of the tea genome and tea quality. Proc. Natl. Acad. Sci. USA.

[22] Qiao D., Yang C., Chen J., Guo Y., Li Y., Niu S., Cao K., Che Z. (2019). Comprehensive identification of the full-length transcripts and alternative splicing related to the secondary metabolism pathways in the tea plant (*Camellia sinensis*). Sci. Rep..

[23] Mondal T.K., Bhattacharya A., Ahuja P.S., Chand P.K. (2001). Factor effecting *Agrobacterium tumefaciens* mediated transformation of tea (*Camellia sinensis* (L). O. Kuntze. Plant Cell Rep..

[24] Osato N., Yamada H., Satoh K., Ooka H., Yamamoto M., Suzuki K., Kawai J., Carninci P., Ohtomo Y., Murakami K. (2003). Antisense transcripts with rice full-length cDNAs. Genome Biol..

[25] Brandle J.E., Richman A., Swanson A.K., Chapman B.P. (2002). Leaf ESTs from Stevia rebaudiana: A resource for gene discovery in diterpene synthesis. Plant Mol. Biol..

[26] Zhu J.Y., Wang X.W., Xu Q.S., Zhao S.Q., Tai Y.L., Wei C.L. (2018). Global dissection of alternative splicing uncovers transcriptional diversity in tissues and associates with the flavonoid pathway in tea plant (*Camellia sinensis*). BMC Plant Biol..

[27] Srivastava A.K., Lu Y., Zinta G., Lang Z., Zhu J.K. (2018). UTR-Dependent Control of Gene Expressionin Plants. Trends Plant Sci..

[28] Pang P.P., Meyerowitz E.M. (1987). Arabidopsis thaliana: A model system for plant molecular biology. Nat. Biotechnol..

[29] Taji T., Ohsumi C., Iuchi S., Seki M., Kasuga M., Kobayashi M., Yamaguchi-Shinozaki K., Shinozaki K. (2002). Important roles of drought- and cold-inducible genes for galactinol synthase in stress tolerance in Arabidopsis thaliana. Plant J..

[30] Andrés-Colás N., Sancenón V., Rodríguez-Navarro S., Mayo S., Thiele D.J., Ecker J.R., Puig S., Peń arrubia L. (2006). The Arabidopsis heavy metal P-type ATPase HMA5 interacts with metallochaperones and functions in copper detoxification of roots. Plant. J..

[31] Lucila G., Elina W., Uta G., Agustín L.A., Iris S., Daniel H.G. (2016). The cytochrome c oxidase biogenesis factor AtCOX17 modulates stress responses in Arabidopsis. Plant Cell Environ..

[32] Attallah C.V., Welchen E., Gonzalez D.H. (2007). The promoters of Arabidopsis thaliana genes AtCOX17-1 and -2, encoding a copper chaperone involved in cytochrome c oxidase biogenesis, are preferentially active in roots and anthers and induced by biotic and abiotic stress. Physiol. Plant..

[33] Klaumann S., Nickolaus S.D., Fürst S.H., Starck S., Schneider S., Ekkehard Neuhaus H., Trentmann O. (2011). The tonoplast copper transporter COPT5 acts as an exporter and is required for interorgan allocation of copper in Arabidopsis thaliana. New Phytol..

[34] Binder B.M., Rodríguez F.I., Bleecker A.B. (2010). The Copper Transporter RAN1 Is Essential for Biogenesis of Ethylene Receptors in Arabidopsis. J. Biol. Chem..

[35] Abdel-Ghany S.E., Müller-Moulé P., Niyogi K.K., Pilon M., Shikanai T. (2005). Two P-Type ATPases Are Required for Copper Delivery inArabidopsis thaliana Chloroplasts. Plant Cell.

[36] Li Y., Iqbal M., Zhang Q., Spelt C., Bliek M., Hakvoort H.W.J., Quattrocchio F.M., Koes R., Schat H. (2017). Two Silene vulgaris copper transporters residing in different cellular compartments confer copper hypertolerance by distinct mechanisms when expressed in Arabidopsis thaliana. New Phytol..

[37] Porebski S., Bailey L.G., Baum B.R. (1997). Modification of a CTAB DNA extraction protocol for plants containing high polysaccharide and polyphenol components. Plant Mol. Biol. Rep..

[38] Xia E., Li F., Tong W., Li P., Wu Q., Zhao H., Ge R., Li R., Li Y., Zhang Z. (2019). Tea plant information archive (TPIA): A comprehensive genomics and bioinformatics platform for tea plant. Plant Biotechnol. J..

[39] Livak K.J., Schmittgen T.D. (2001). Analysis of relative gene expression data using real-time quantitative PCR and the 2^−ΔΔ*C*t^ method. Methods.

[40] Carninci P., Kvam C., Kitamura A., Ohsumi T., Okazaki Y., Itoh M., Kamiya M., Shibata K., Sasaki N., Izawa M. (1996). High-efficiency full-length cDNA cloning by biotinylated CAP trapper. Genomics.

[41] Edery I., Chu L., Sonenberg N., Pelletier J. (1995). An efficient strategy to isolate full-length cDNAs based on an mRNA cap retention procedure (CAPture). Mol. Cell Biol..

[42] Zhu Y.Y., Machleder E.M., Chenchik A., Li R., Siebert P.D. (2001). Reverse transcriptase template switching: A SMART (TM) approach for full-length cDNA library construction. Biotechniques.

[43] Belyavsky A., Vinogradova T.V., Rajewsky K. (1989). PCR-based cDNA library construction: General cDNA libraries at the level of a few cells. Nucl. Acids Res..

[44] Carninci P., Shibata Y., Hayatsu N., Itoh M., Shiraki T., Hirozane T., Watahiki A., Shibata K., Konno H., Muramatsu M. (2001). Balanced-size and long-size cloning of full-length, cap-trapped cDNAs into vectors of the novel lambda-FLC family allows enhanced gene discovery rate and functional analysis. Genomics.

[45] Ichikawa T., Nakazawa M., Kawashima M., Iizumi H., Kuroda H., Kondou Y., Tsuhara Y., Suzuki K., Ishikawa A., Seki M. (2006). The FOX hunting system: An alternative gain-of-function gene hunting technique. Plant. J..

[46] Kondou Y., Higuchi M., Takahashi S., Sakurai T., Ichikawa T., Kuroda H., Yoshizumi T., Tsumoto Y., Horii Y., Kawashima M. (2009). Systematic approaches to using the FOX hunting system to identify useful rice genes. Plant. J..

