# Supplementary material for: Establishing a System for Functional Characterization of Full-Length cDNAs of Camellia sinensis
Source: Int J Mol Sci. 2019 Nov 25;20(23):5929. doi: 10.3390/ijms20235929 (PMC6929147; doi:10.3390/ijms20235929)
Supplement: Supplementary file 1 [file ijms-20-05929-s001.zip › ijms-647714-suppl/Figure S1-S13.pptx]

## Slide 1
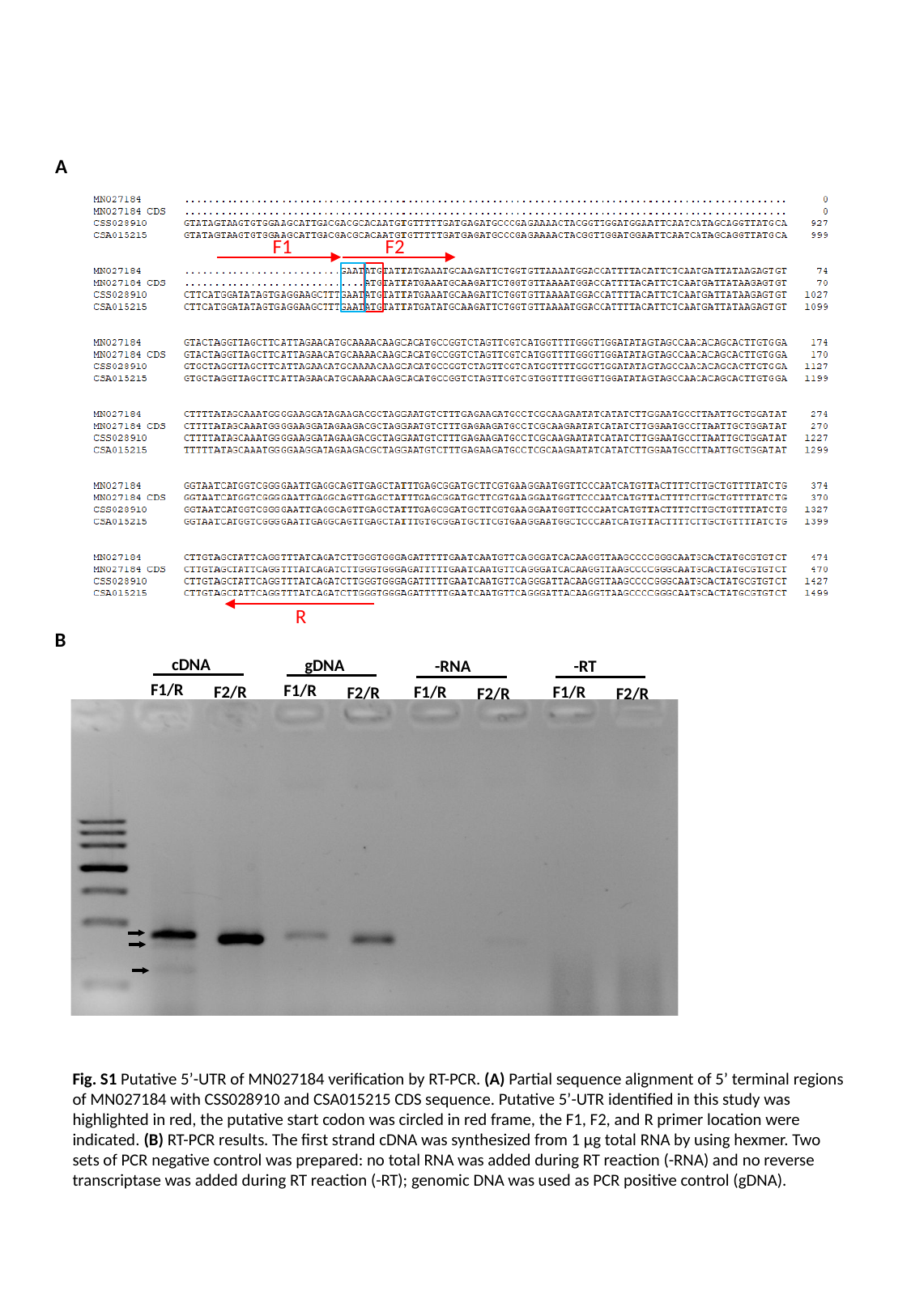

A
F2
F1
R
B
 cDNA
 gDNA
 -RNA
 -RT
F1/R
F1/R
F2/R
F1/R
F1/R
F2/R
F2/R
F2/R
Fig. S1 Putative 5’-UTR of MN027184 verification by RT-PCR. (A) Partial sequence alignment of 5’ terminal regions of MN027184 with CSS028910 and CSA015215 CDS sequence. Putative 5’-UTR identified in this study was highlighted in red, the putative start codon was circled in red frame, the F1, F2, and R primer location were indicated. (B) RT-PCR results. The first strand cDNA was synthesized from 1 µg total RNA by using hexmer. Two sets of PCR negative control was prepared: no total RNA was added during RT reaction (-RNA) and no reverse transcriptase was added during RT reaction (-RT); genomic DNA was used as PCR positive control (gDNA).

## Slide 2
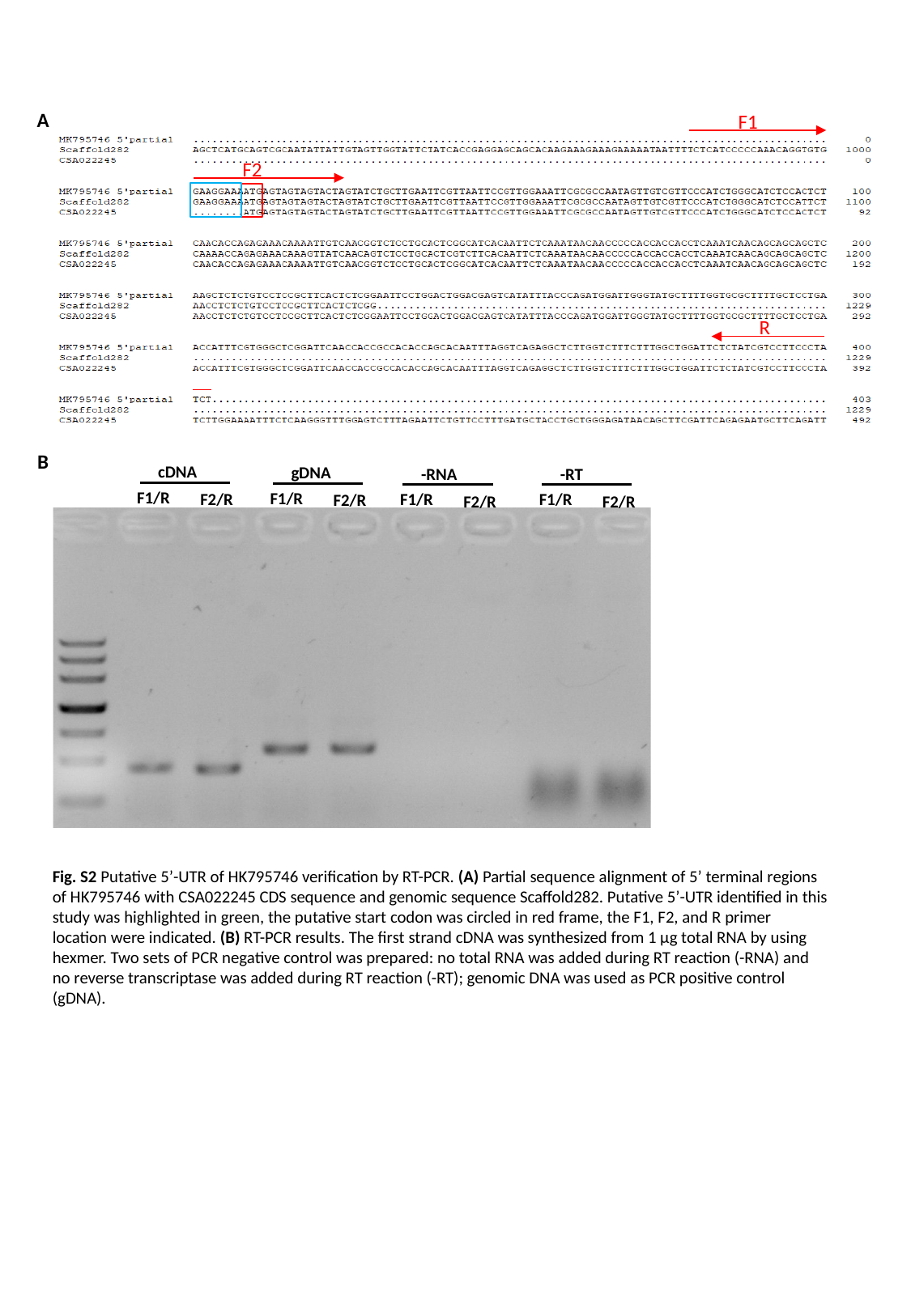

A
F1
F2
R
B
 cDNA
 gDNA
 -RNA
 -RT
F1/R
F1/R
F2/R
F1/R
F1/R
F2/R
F2/R
F2/R
Fig. S2 Putative 5’-UTR of HK795746 verification by RT-PCR. (A) Partial sequence alignment of 5’ terminal regions of HK795746 with CSA022245 CDS sequence and genomic sequence Scaffold282. Putative 5’-UTR identified in this study was highlighted in green, the putative start codon was circled in red frame, the F1, F2, and R primer location were indicated. (B) RT-PCR results. The first strand cDNA was synthesized from 1 µg total RNA by using hexmer. Two sets of PCR negative control was prepared: no total RNA was added during RT reaction (-RNA) and no reverse transcriptase was added during RT reaction (-RT); genomic DNA was used as PCR positive control (gDNA).

## Slide 3
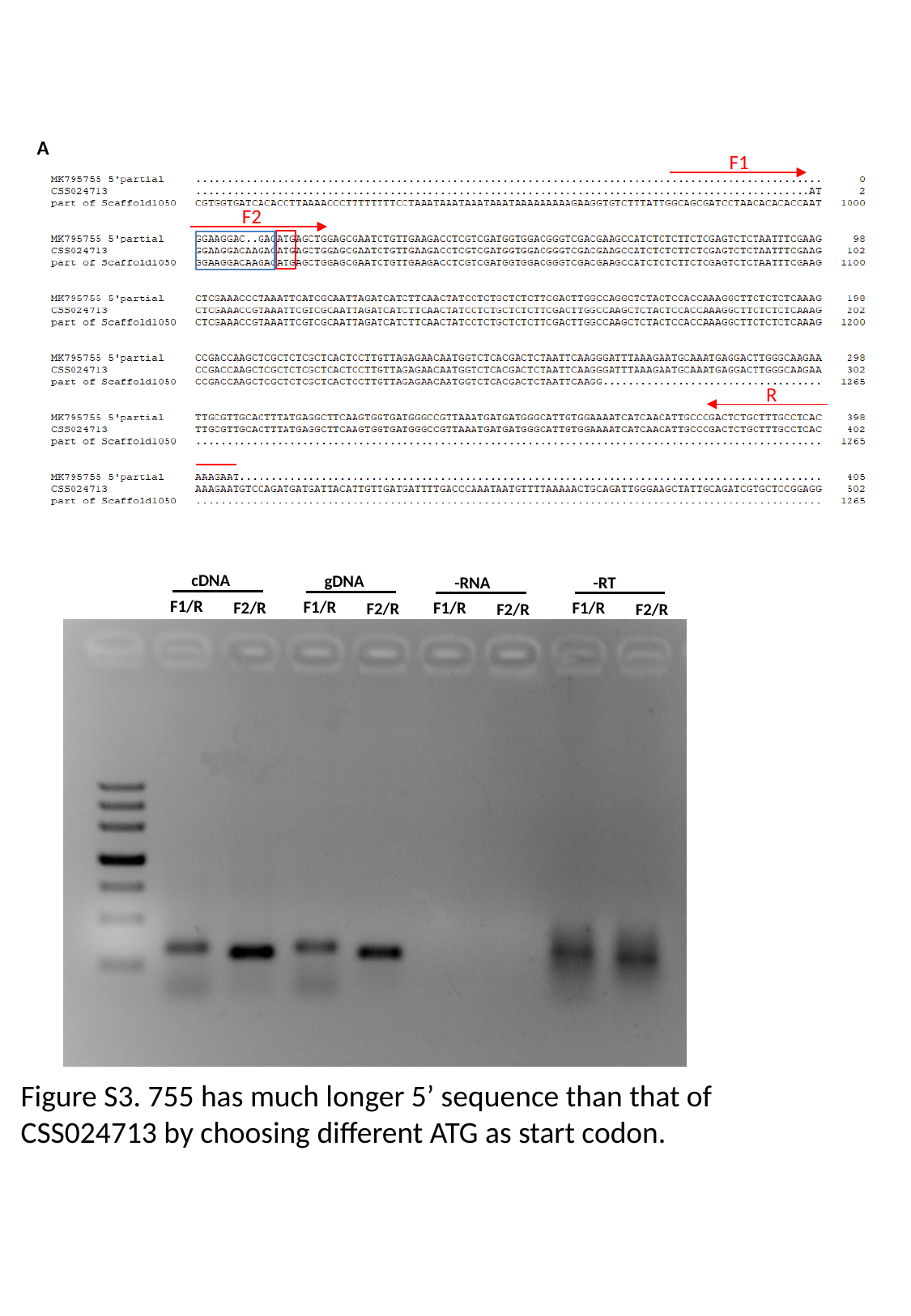

A
F1
F2
R
 cDNA
 gDNA
 -RNA
 -RT
F1/R
F1/R
F2/R
F1/R
F1/R
F2/R
F2/R
F2/R
Figure S3. 755 has much longer 5’ sequence than that of CSS024713 by choosing different ATG as start codon.

## Slide 4
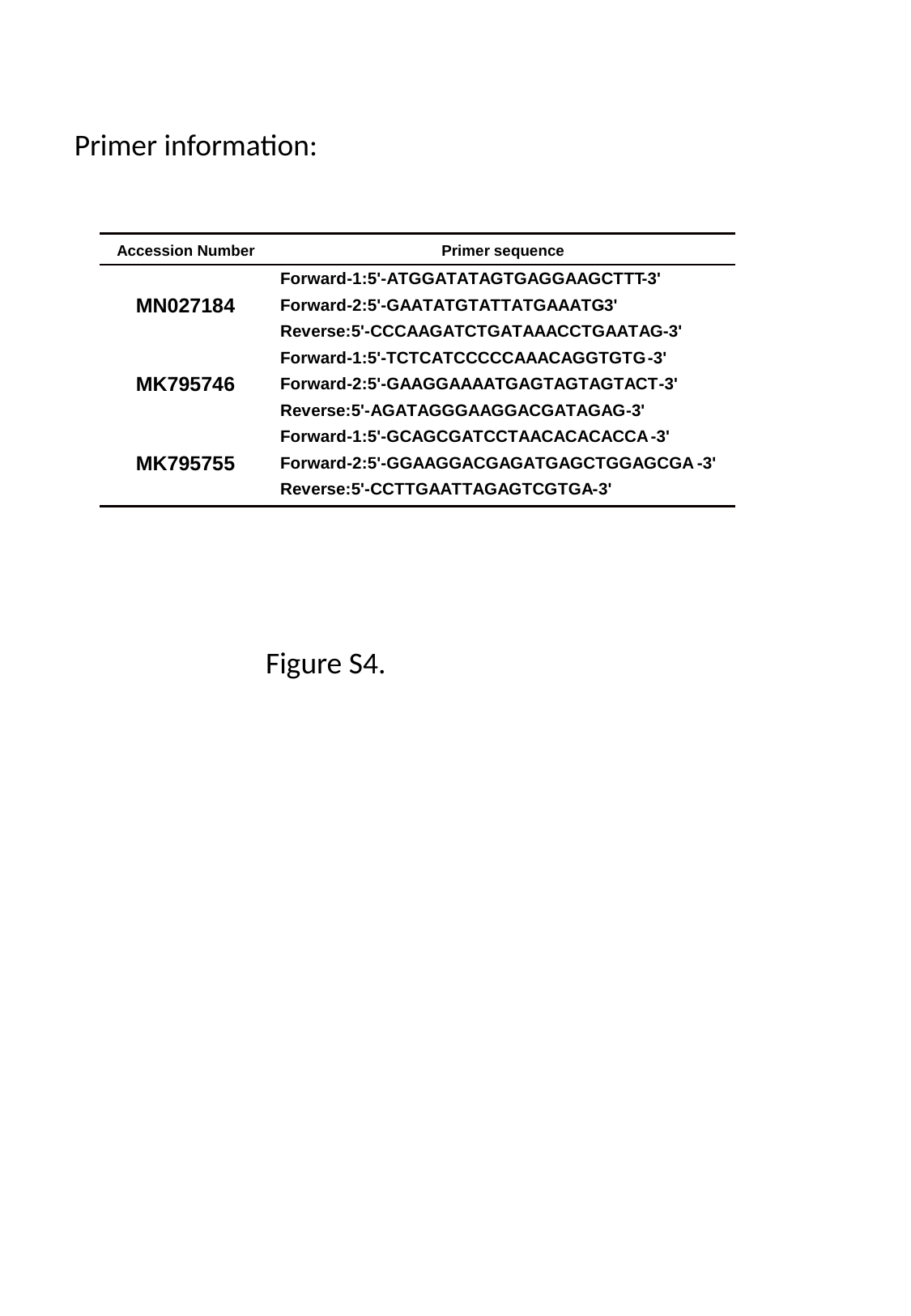

Primer information:
Figure S4.

## Slide 5
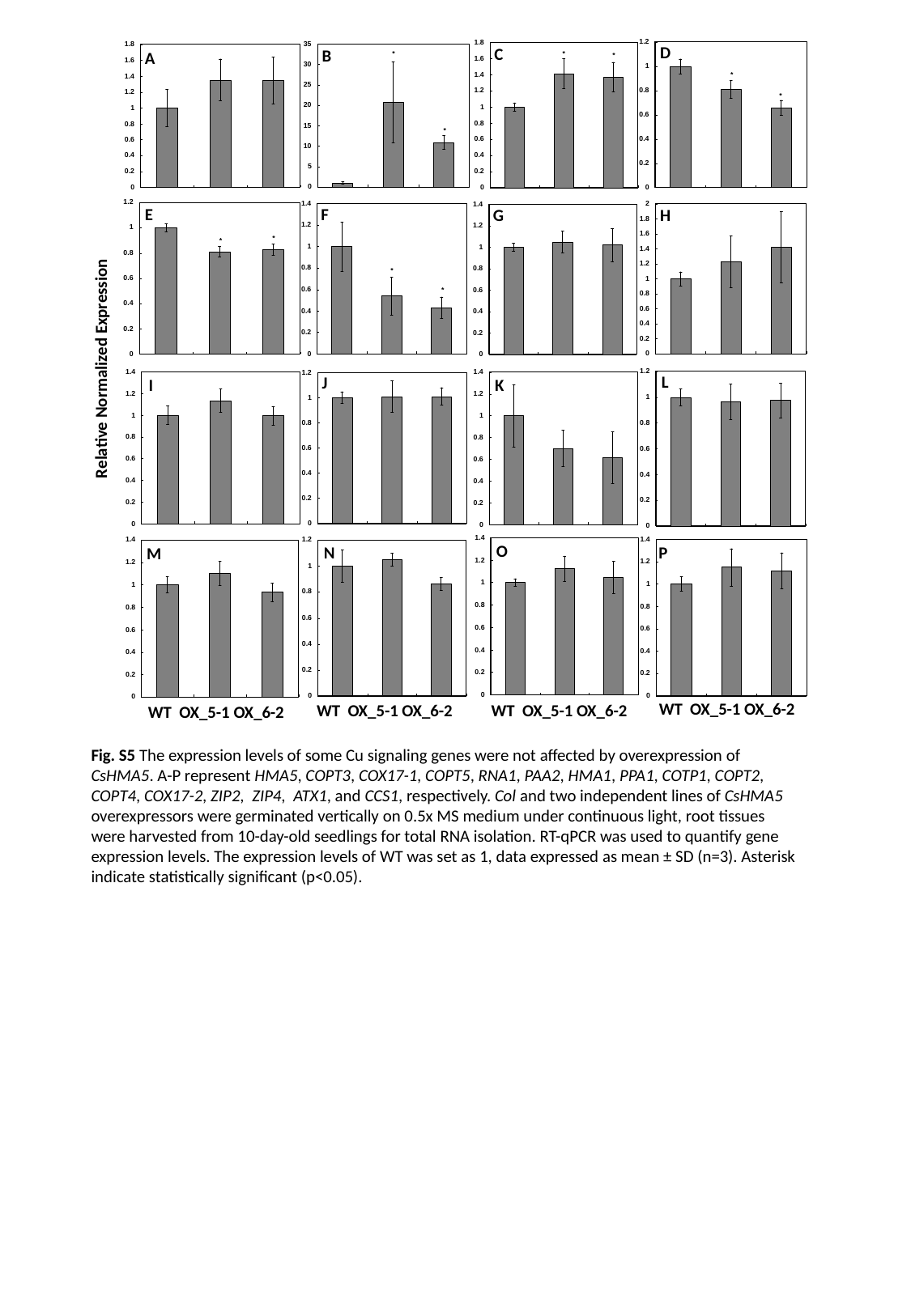

D
C
B
A
E
F
G
H
Relative Normalized Expression
L
J
I
K
O
N
P
M
WT OX_5-1 OX_6-2
WT OX_5-1 OX_6-2
WT OX_5-1 OX_6-2
WT OX_5-1 OX_6-2
Fig. S5 The expression levels of some Cu signaling genes were not affected by overexpression of CsHMA5. A-P represent HMA5, COPT3, COX17-1, COPT5, RNA1, PAA2, HMA1, PPA1, COTP1, COPT2, COPT4, COX17-2, ZIP2, ZIP4, ATX1, and CCS1, respectively. Col and two independent lines of CsHMA5 overexpressors were germinated vertically on 0.5x MS medium under continuous light, root tissues were harvested from 10-day-old seedlings for total RNA isolation. RT-qPCR was used to quantify gene expression levels. The expression levels of WT was set as 1, data expressed as mean ± SD (n=3). Asterisk indicate statistically significant (p<0.05).

## Slide 6
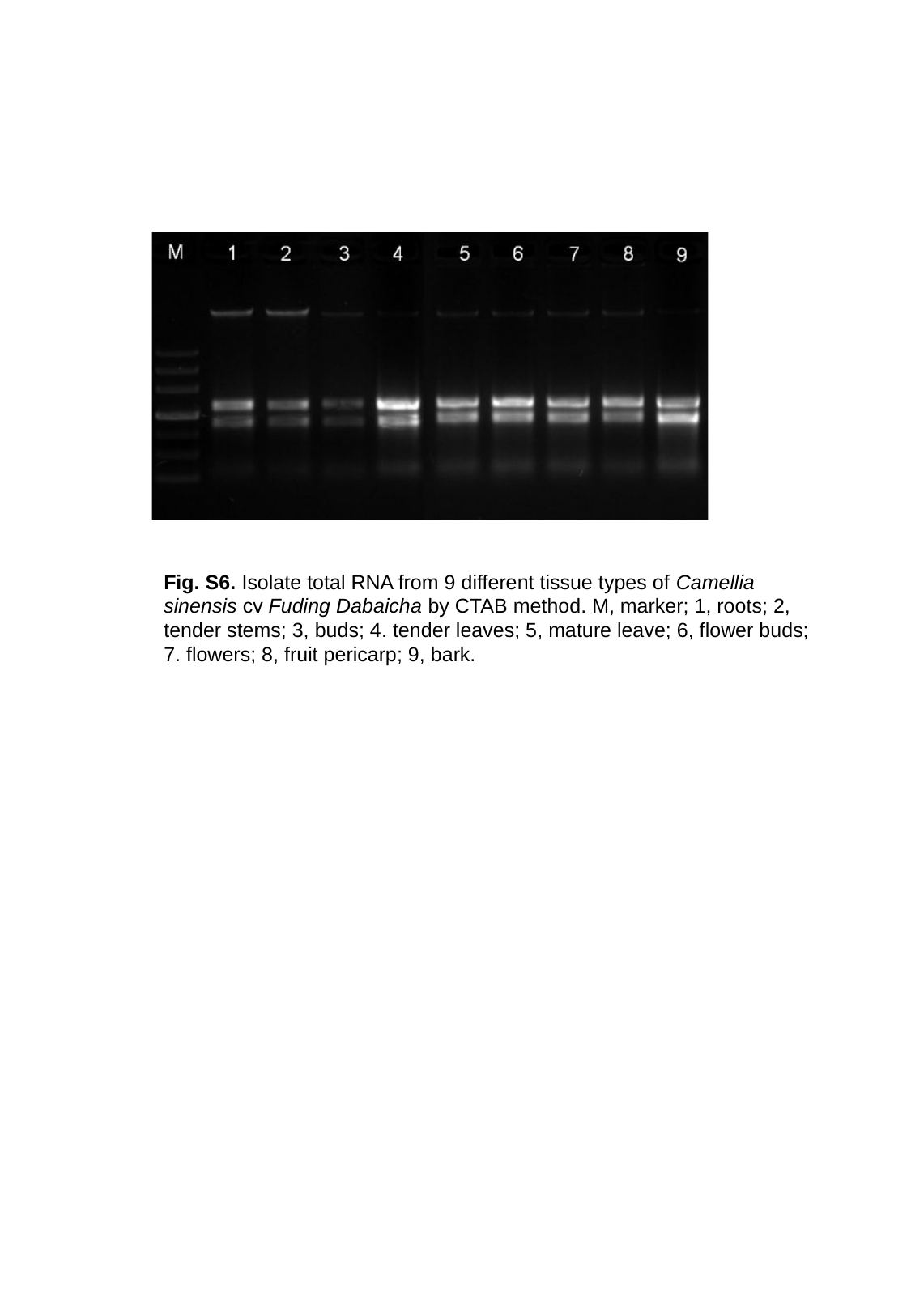

M 1 2 3 4 5 6 7 8 9
Fig. S6. Isolate total RNA from 9 different tissue types of Camellia sinensis cv Fuding Dabaicha by CTAB method. M, marker; 1, roots; 2, tender stems; 3, buds; 4. tender leaves; 5, mature leave; 6, flower buds; 7. flowers; 8, fruit pericarp; 9, bark.

## Slide 7
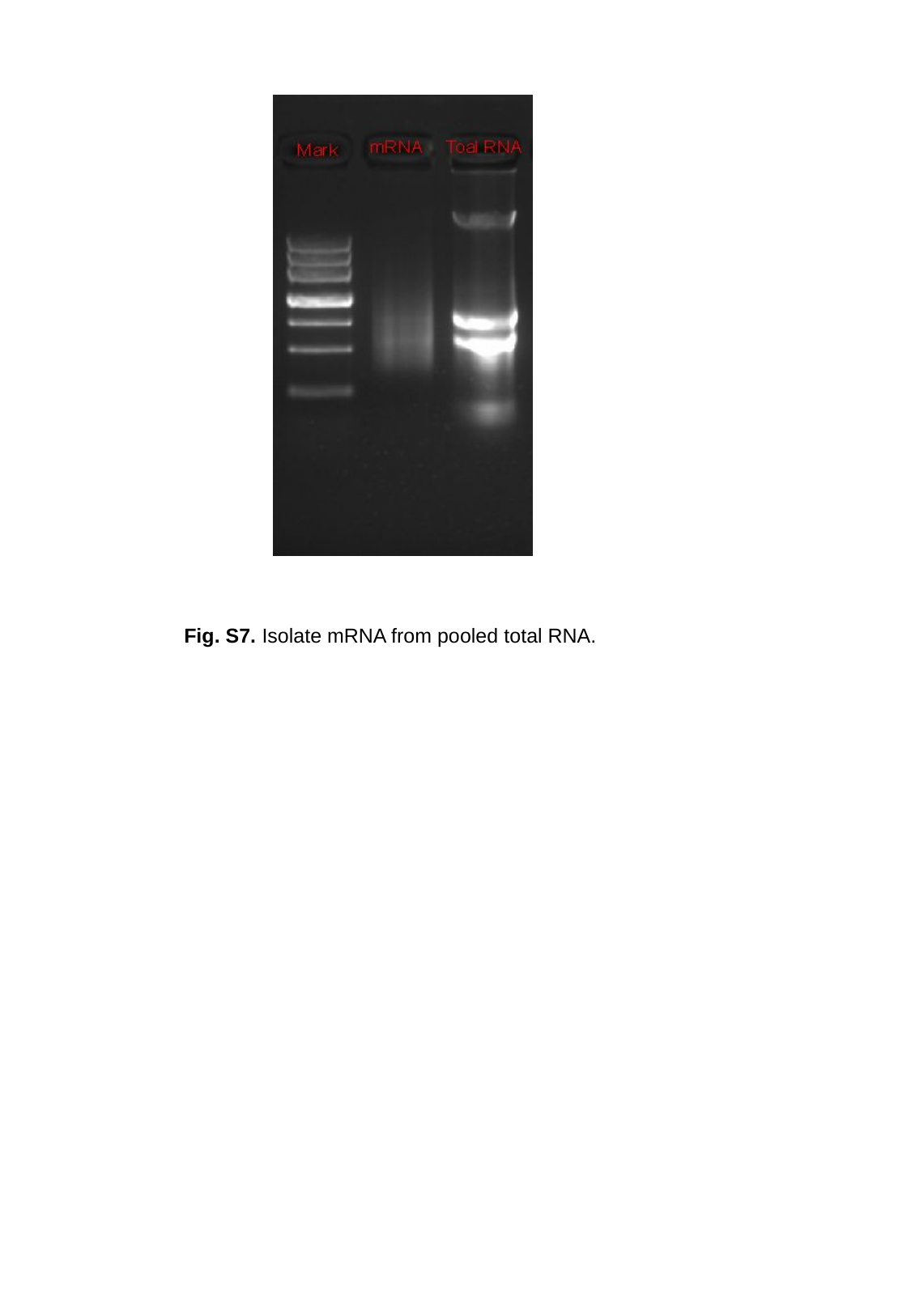

Fig. S7. Isolate mRNA from pooled total RNA.

## Slide 8
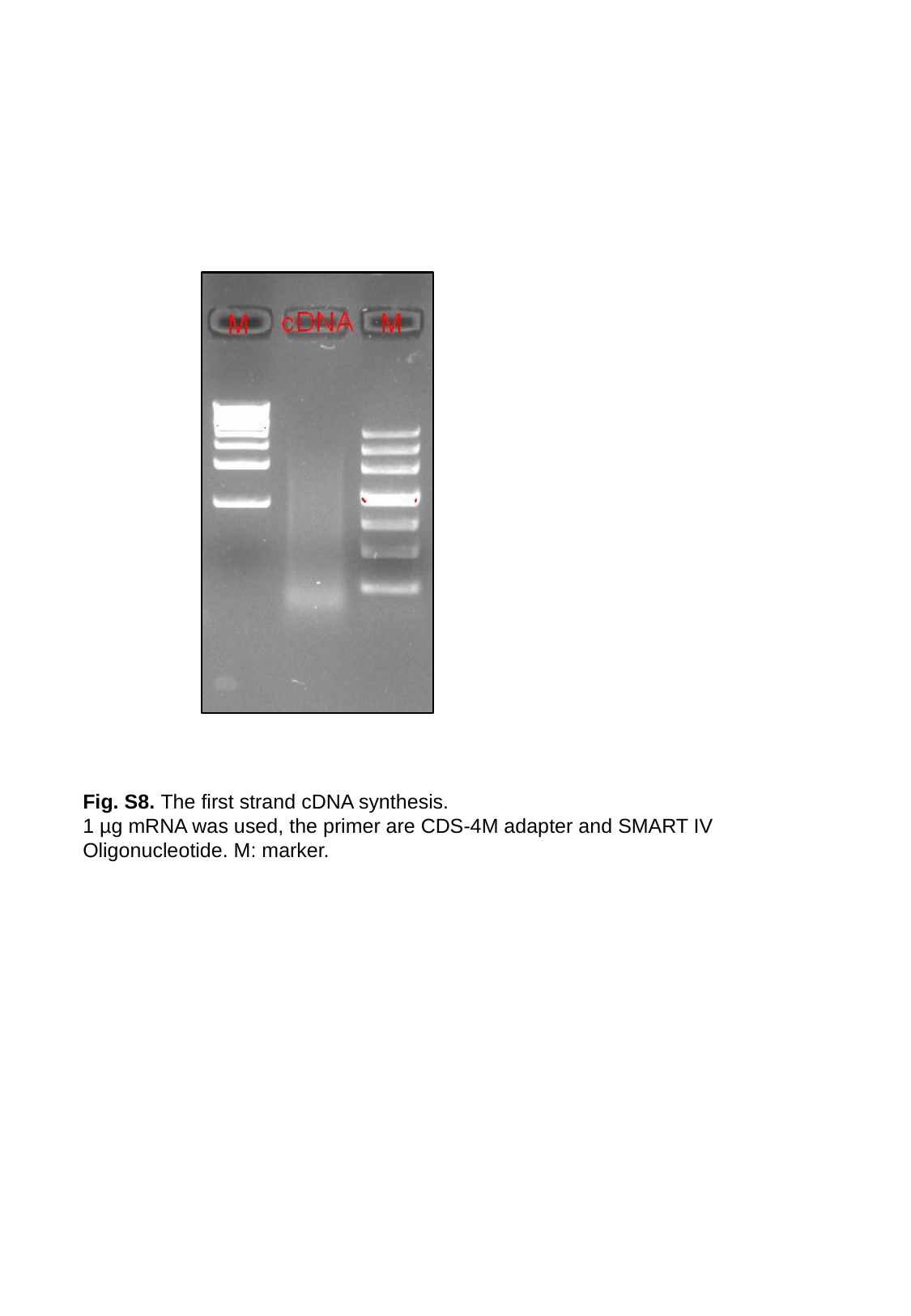

Fig. S8. The first strand cDNA synthesis.
1 µg mRNA was used, the primer are CDS-4M adapter and SMART IV Oligonucleotide. M: marker.

## Slide 9
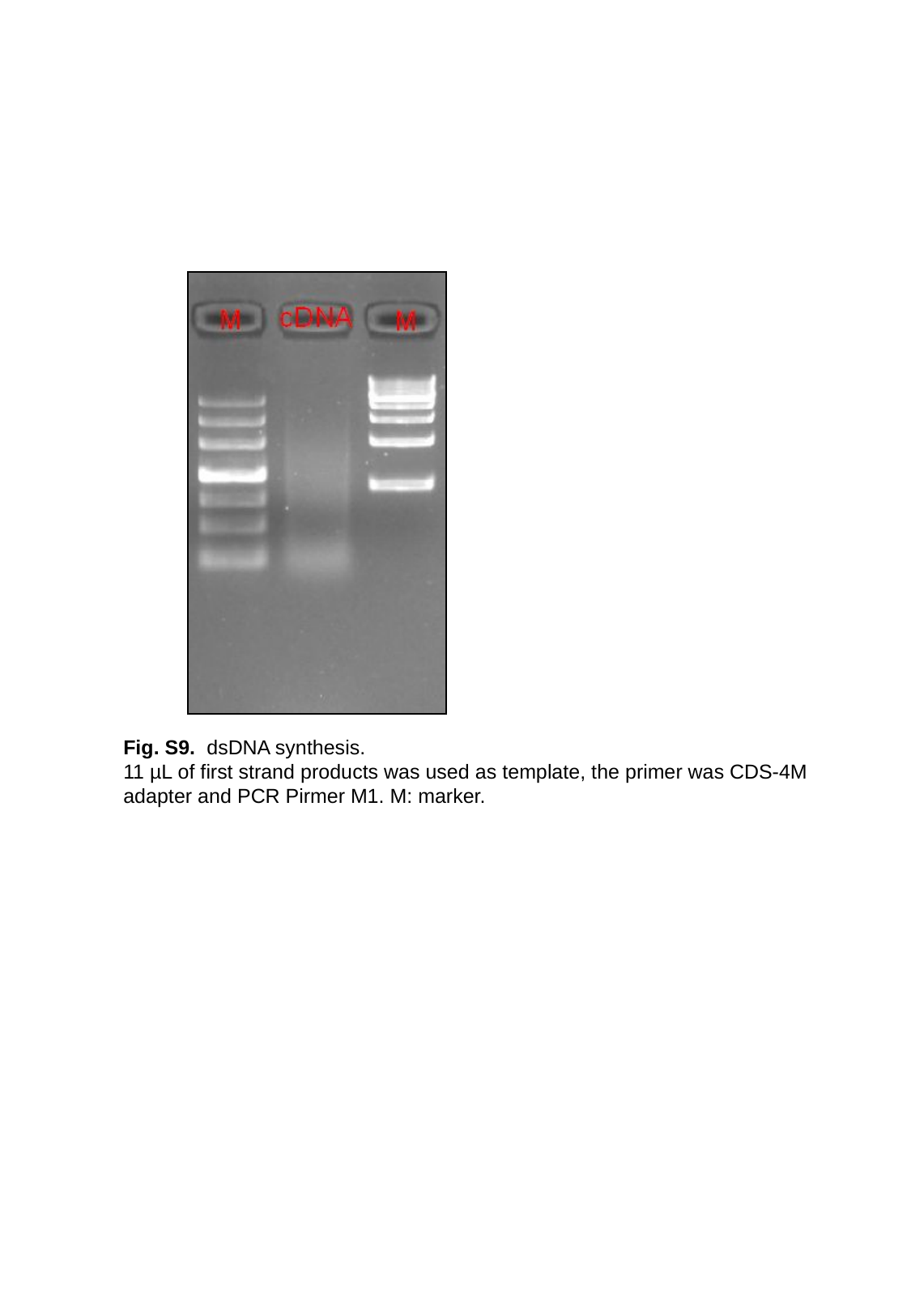

Fig. S9. dsDNA synthesis.
11 µL of first strand products was used as template, the primer was CDS-4M adapter and PCR Pirmer M1. M: marker.

## Slide 10
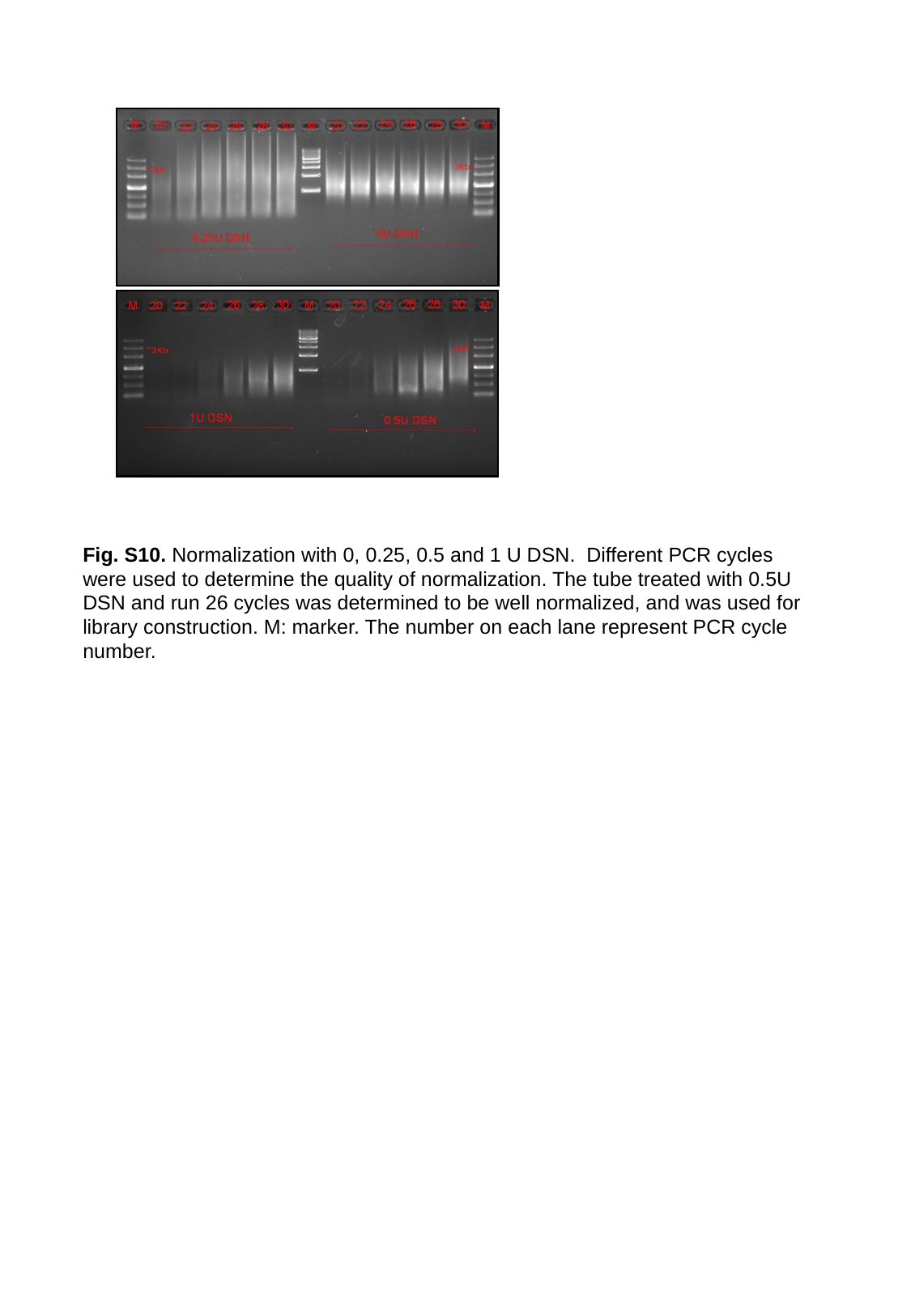

Fig. S10. Normalization with 0, 0.25, 0.5 and 1 U DSN. Different PCR cycles were used to determine the quality of normalization. The tube treated with 0.5U DSN and run 26 cycles was determined to be well normalized, and was used for library construction. M: marker. The number on each lane represent PCR cycle number.

## Slide 11
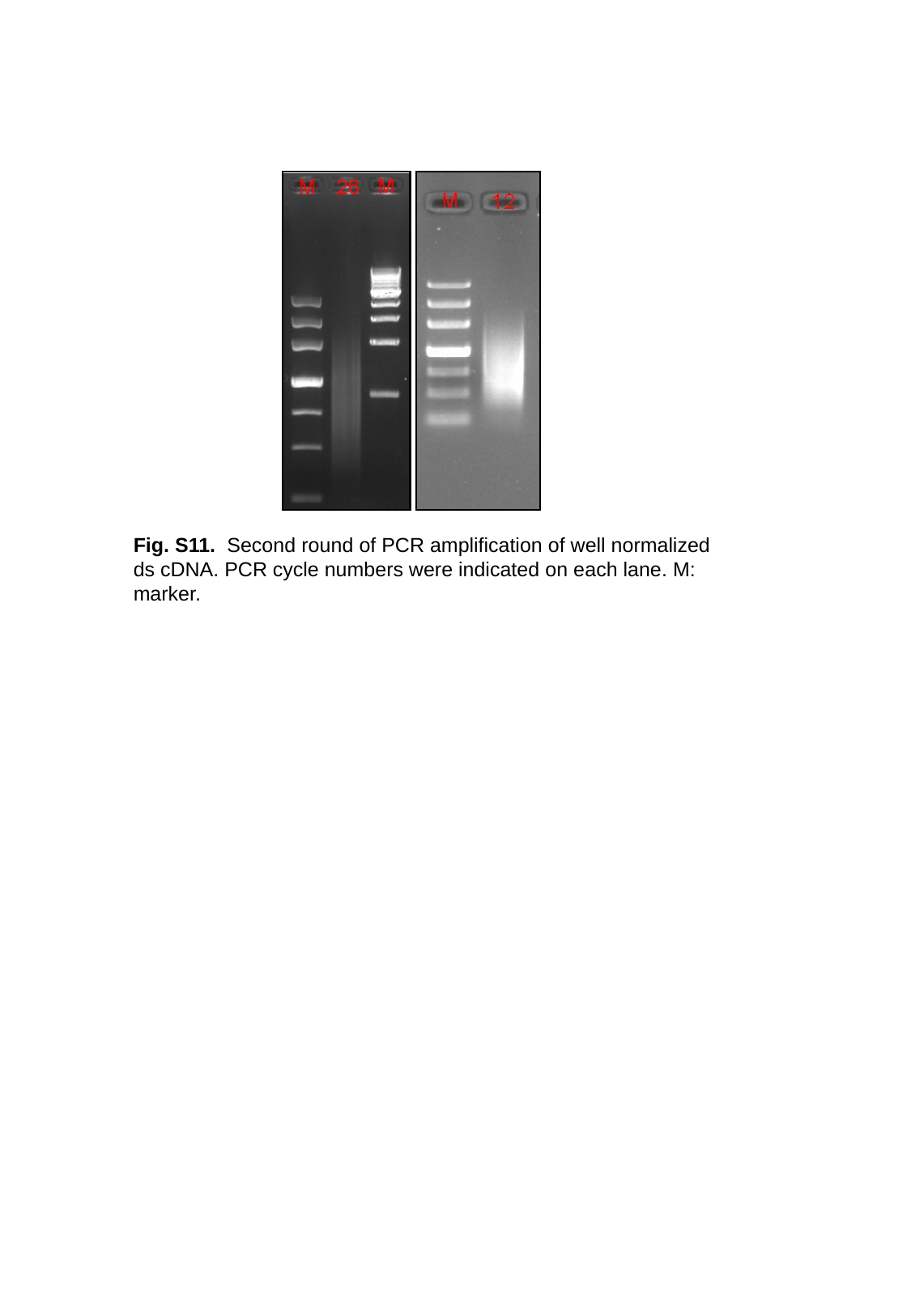

Fig. S11. Second round of PCR amplification of well normalized ds cDNA. PCR cycle numbers were indicated on each lane. M: marker.

## Slide 12
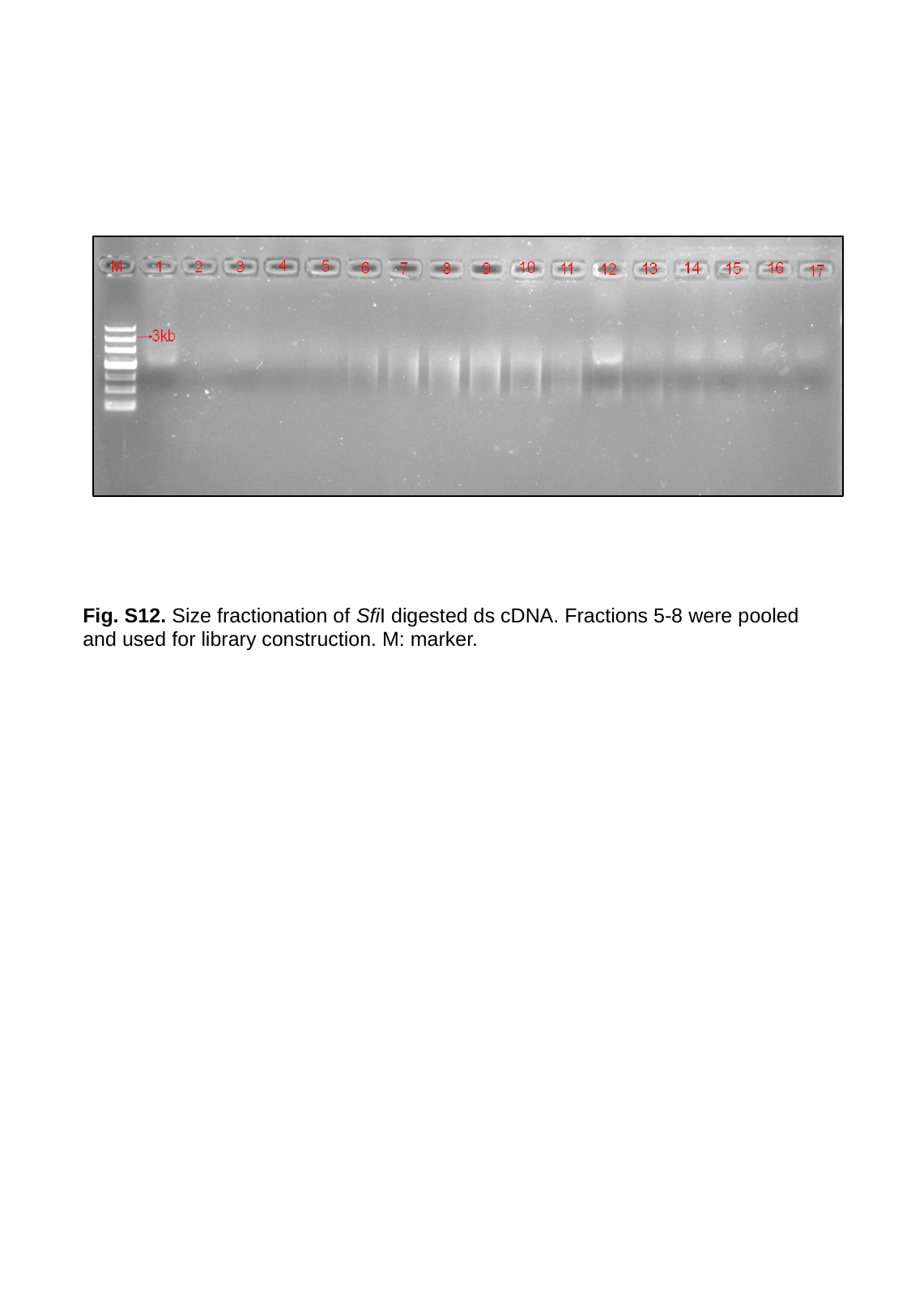

Fig. S12. Size fractionation of SfiI digested ds cDNA. Fractions 5-8 were pooled and used for library construction. M: marker.

## Slide 13
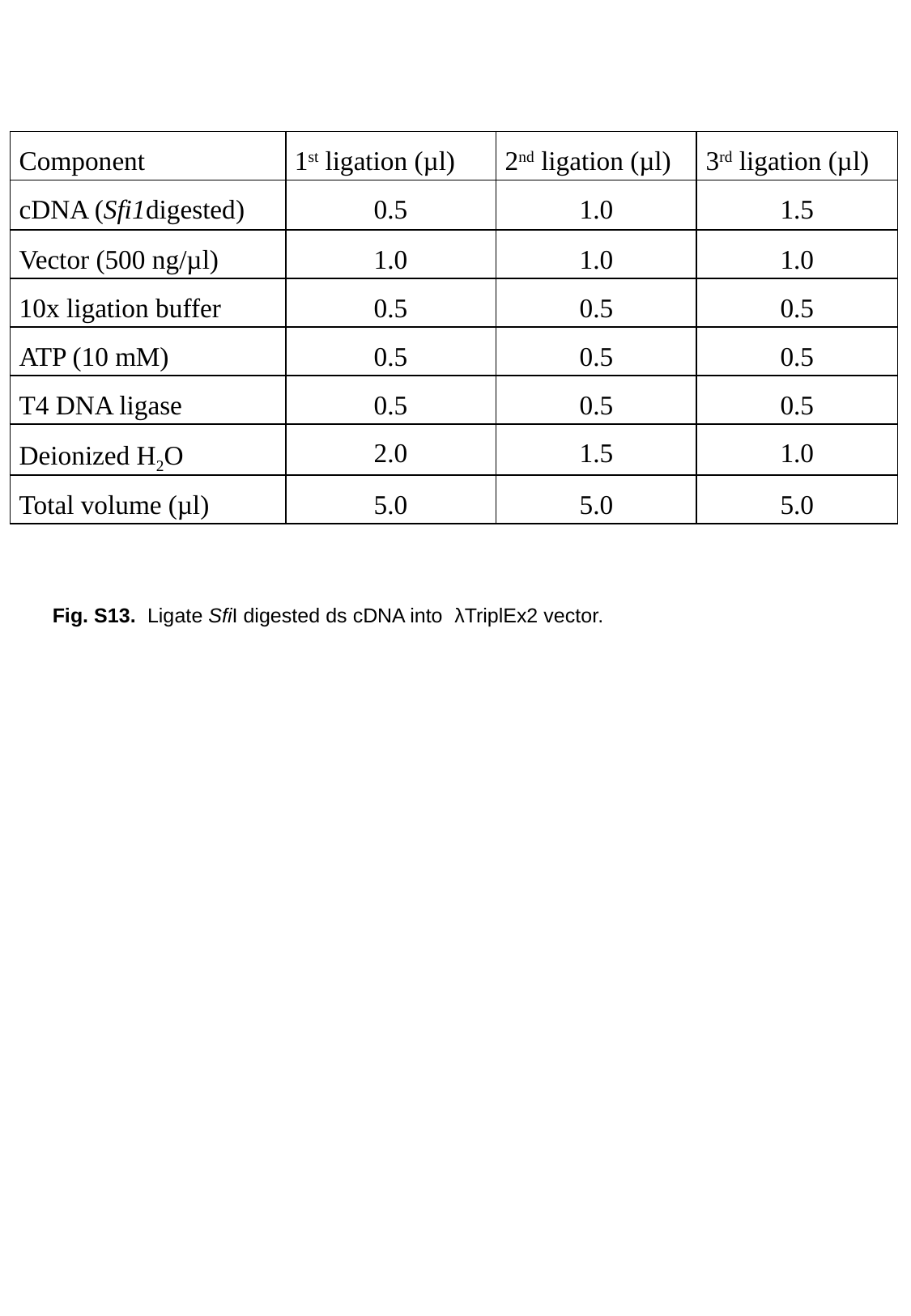

| Component | 1st ligation (µl) | 2nd ligation (µl) | 3rd ligation (µl) |
| --- | --- | --- | --- |
| cDNA (Sfi1digested) | 0.5 | 1.0 | 1.5 |
| Vector (500 ng/µl) | 1.0 | 1.0 | 1.0 |
| 10x ligation buffer | 0.5 | 0.5 | 0.5 |
| ATP (10 mM) | 0.5 | 0.5 | 0.5 |
| T4 DNA ligase | 0.5 | 0.5 | 0.5 |
| Deionized H2O | 2.0 | 1.5 | 1.0 |
| Total volume (µl) | 5.0 | 5.0 | 5.0 |
Fig. S13. Ligate SfiI digested ds cDNA into λTriplEx2 vector.
