# Supplementary material for: Establishing a System for Functional Characterization of Full-Length cDNAs of Camellia sinensis
Source: Int J Mol Sci. 2019 Nov 25;20(23):5929. doi: 10.3390/ijms20235929 (PMC6929147; doi:10.3390/ijms20235929)
Supplement: Supplementary file 1 [file ijms-20-05929-s001.zip › ijms-647714-suppl/Table S1 and S2.pdf]

Table S1. Primer sequence of tea clone 2, 8, 9, 12, 21, 27, 28.

Clone Number	Gene Bank Accession Number	Forward/Reverse Primer Sequence(5′-3′)	CDS Length (bp)
**2**	MK795745	ATGGAGAACGCCGACGTGTTTGGCTCGT<br>TGATGCTCTTTGTATTCAATGCTCTTGT	868
**8**	MK795749	ATGATGAATGAAAACATTTTCAT<br>TCAAGCTATTGGGTCTTTGTAGT	684
**9**	MN027185	ATGAAAACACAGCATCTTGATCTCAGT<br>TTACCTTCTGGATAAGAGTATCATCG	690
**12**	MK795751	ATGGGTTGTTTTTGTTGTAAGCCCTCGCTAT<br>CTAAGGCTGTGCAGGGAAGTCGTTAAATGTAT	879
**21**	MK795756	ATGGGCTCCCAAACAGTTCCCAAGCTTCCTGTTGTA<br>TTACTTCTCTTTCCCATTCTCTTGGGGTGGCGTG	768
**27**	MK795760	ATGTTACGGTTCACACTTCGACGAGGAGGAGCGA<br>TCACATTGCATCAGCATGACAACTCATGAT	900
**28**	MK795761	ATGGGTGGGTGTGAAGCAAACGA<br>TTAATATTTGCTGGGTGGGGTTGAT	516

Table S2 Primer sequences of Cu signaling components in Arabidopsis

Gene Name	Accession Number	Primer sequence
COPT1	AT5G59030	Forward: 5'-GTTAATCCAAACCGCCGTGTA-3'<br>Reverse: 5'-CAGAGCGACGAGAAACACACC-3'
COPT2	AT3G46900	Forward: 5'-TCGCTCAAACCGCTGTGTAC-3'<br>Reverse: 5'-AGAGAAAGAAACCAACGCCATAG-3'
COPT3	AT5G59040	Forward: 5'-CCTTCACCATCATCGTTCTTCC-3'<br>Reverse: 5'-CGGCGAGACAGACCCAATAC-3'
COPT4	AT2G37925	Forward: 5'-CTGACCGTGGGATGTATGCA-3'<br>Reverse: 5'-TCGGCACCCTGTTTGATG-3'
COPT5	AT5G20650	Forward: 5'-GAGAATCGCCGCATCCAAT-3'<br>Reverse: 5'-TGACGCCGAAAAGAAGAACC-3'
ZIP2	AT5G59520	Forward: 5'-GTACGTTGCGGTTAACCATCTC-3'<br>Reverse: 5'-CGAGGAAGACGGCAATAAACTT-3'
ZIP4	AT1G10970	Forward: 5'-GCTCACCATAGGCATAGTCACTCT-3'<br>Reverse: 5-CACAATCCCGAGCTCCAATAT-3'
COX17-1	AT3G15352	Forward: 5'-GATTGATTCCTCCACCCACTTC-3'<br>Reverse: 5'-ACATATCCTCTTCTTTGGTTTCGTC-3'
COX17-2	AT1G53030	Forward: 5'-GGACAAGCCAAGCAAAGATGT-3'<br>Reverse: 5'-GATTCACCGTGTTCTACAATGCA-3'
ATX1	AT1G66240	Forward: 5'-TGTTCCAAGCCGTATCCTATCA-3'<br>Reverse: 5'-TCCACGCCTTCCATTTTCC-3'
CCS1	AT1G12520	Forward: 5'-AGCAAACTGGTCGAAAAGCTC-3'<br>Reverse: 5'-GGGCCTTTGAATTCTGCTACTG-3'
HMA1	AT4G37270	Forward: 5'-TGGCCGCTCTTCCTTCAG-3'<br>Reverse:5'-GAGCGTAACTTGTTGATTAGATGAACT-3'
HMA5	AT1G63440	Forward: 5'-GGAAGCCCGTTGTTGTGA-3'<br>Reverse: 5'-CCTTTGCTAACGGATGCTCACT-3'
HMA6/PAA1	AT4G33520	Forward: 5'-GTGGGCGTTCGGATACAAC-3'<br>Reverse: 5'-CTCTTTTGGTTCCGGTTTGAC-3'
HMA7/RAN1	AT5G44790	Forward: 5'-TGGTAGGAGACGGAATCAATGAC-3'<br>Reverse: 5'-CGATGGCTGTTATAACGTCTTCTAAG-3'
HMA8/PAA2	AT5G21930	Forward: 5'-GCTTTGTGCTCCTTGGTCGT-3'<br>Reverse: 5'-CAGAATCCACTGGGGTGTTATTG-3'
AP2M	AT5G46630	Forward: 5'-TCGATTGCTTGGTTTGGAAGAT-3'<br>Reverse: 5'-TGTCCAAGATTTCTTCTCTCCCAT-3'
