# Supplementary material for: Establishing a System for Functional Characterization of Full-Length cDNAs of Camellia sinensis
Source: Int J Mol Sci. 2019 Nov 25;20(23):5929. doi: 10.3390/ijms20235929 (PMC6929147; doi:10.3390/ijms20235929)
Supplement: Supplementary file 1 [file ijms-20-05929-s001.zip › Table S1.docx]

**Table S1.** Primer sequence of tea clone 2, 8, 9, 12, 21, 27, 28

| **Clone Number** | **Gene Bank**  **Accession Number** | **Forward/Reverse Primer**  **Sequence(5’-3’)** | **CDS Length**  **(bp)** |
| --- | --- | --- | --- |
| **2** | MK795745 | ATGGAGAACGCCGACGTGTTTGGCTCGT  TGATGCTCTTTGTATTCAATGCTCTTGT | 868 |
| **8** | MK795749 | ATGATGAATGAAAACATTTTCAT  TCAAGCTATTGGGTCTTTGTAGT | 684 |
| **9** | MN027185 | ATGAAAACACAGCATCTTGATCTCAGT  TTACCTTCTGGATAAGAGTATCATCG | 690 |
| **12** | MK795751 | ATGGGTTGTTTTTGTTGTAAGCCCTCGCTAT  CTAAGGCTGTGCAGGGAAGTCGTTAAATGTAT | 879 |
| **21** | MK795756 | ATGGGCTCCCAAACAGTTCCCAAGCTTCCTGTTGTA  TTACTTCTCTTTCCCATTCTCTTGGGGTGGCGTG | 768 |
| **27** | MK795760 | ATGTTACGGTTCACACTTCGACGAGGAGGAGCGA  TCACATTGCATCAGCATGACAACTCATGAT | 900 |
| **28** | MK795761 | ATGGGTGGGTGTGAAGCAAACGA  TTAATATTTGCTGGGTGGGGTTGAT | 516 |
