# Supplementary material for: Establishing a System for Functional Characterization of Full-Length cDNAs of Camellia sinensis
Source: Int J Mol Sci. 2019 Nov 25;20(23):5929. doi: 10.3390/ijms20235929 (PMC6929147; doi:10.3390/ijms20235929)
Supplement: Supplementary file 1 [file ijms-20-05929-s001.zip › Table S2.docx]

**Table S2** Primer sequences of Cu signaling components in Arabidopsis.

| **Gene Name** | **Accession Number** | **Primer sequence** |
| --- | --- | --- |
| ***COPT1*** | AT5G59030 | Forward: 5'-GTTAATCCAAACCGCCGTGTA-3' |
|  |  | Reverse: 5'-CAGAGCGACGAGAAACACACC-3' |
| ***COPT2*** | AT3G46900 | Forward: 5'-TCGCTCAAACCGCTGTGTAC-3' |
|  |  | Reverse: 5'-AGAGAAAGAAACCAACGCCATAG-3' |
| ***COPT3*** | AT5G59040 | Forward: 5'-CCTTCACCATCATCGTTCTTCC-3' |
|  |  | Reverse: 5'-CGGCGAGACAGACCCAATAC-3' |
| ***COPT4*** | AT2G37925 | Forward: 5'-CTGACCGTGGGATGTATGCA-3' |
|  |  | Reverse: 5'-TCGGCACCCTGTTTGATG-3' |
| ***COPT5*** | AT5G20650 | Forward: 5'-GAGAATCGCCGCATCCAAT-3' |
|  |  | Reverse: 5'-TGACGCCGAAAAGAAGAACC-3' |
| ***ZIP2*** | AT5G59520 | Forward: 5'-GTACGTTGCGGTTAACCATCTC-3' |
|  |  | Reverse: 5'-CGAGGAAGACGGCAATAAACTT-3' |
| ***ZIP4*** | AT1G10970 | Forward: 5'-GCTCACCATAGGCATAGTCACTCT-3' |
|  |  | Reverse: 5-CACAATCCCGAGCTCCAATAT-3' |
| ***COX17-1*** | AT3G15352 | Forward: 5'-GATTGATTCCTCCACCCACTTC-3' |
|  |  | Reverse: 5'-ACATATCCTCTTCTTTGGTTTCGTC-3' |
| ***COX17-2*** | AT1G53030 | Forward: 5'-GGACAAGCCAAGCAAAGATGT-3' |
|  |  | Reverse: 5'-GATTCACCGTGTTCTACAATGCA-3' |
| ***ATX1*** | AT1G66240 | Forward: 5'-TGTTCCAAGCCGTATCCTATCA-3' |
|  |  | Reverse: 5'-TCCACGCCTTCCATTTTCC-3' |
| ***CCS1*** | AT1G12520 | Forward: 5'-AGCAAACTGGTCGAAAAGCTC-3' |
|  |  | Reverse: 5'-GGGCCTTTGAATTCTGCTACTG-3' |
| ***HMA1*** | AT4G37270 | Forward: 5'-TGGCCGCTCTTCCTTCAG-3' |
|  |  | Reverse:5'-GAGCGTAACTTGTTGATTAGATGAACT-3' |
| ***HMA5*** | AT1G63440 | Forward: 5'-GGAAGCCCGTTGTTGTGA-3' |
|  |  | Reverse: 5'-CCTTTGCTAACGGATGCTCACT-3' |
| ***HMA6/PAA1*** | AT4G33520 | Forward: 5'-GTGGGCGTTCGGATACAAC-3' |
|  |  | Reverse: 5'-CTCTTTTGGTTCCGGTTTGAC-3' |
| ***HMA7/RAN1*** | AT5G44790 | Forward: 5'-TGGTAGGAGACGGAATCAATGAC-3' |
|  |  | Reverse: 5'-CGATGGCTGTTATAACGTCTTCTAAG-3' |
| ***HMA8/PAA2*** | AT5G21930 | Forward: 5'-GCTTTGTGCTCCTTGGTCGT-3' |
|  |  | Reverse: 5'-CAGAATCCACTGGGGTGTTATTG-3' |
| ***AP2M*** | AT5G46630 | Forward: 5'-TCGATTGCTTGGTTTGGAAGAT-3' |
|  |  | Reverse: 5'-TGTCCAAGATTTCTTCTCTCCCAT-3' |
